# Exome sequencing of extended families with autism reveals genes shared across neurodevelopmental and neuropsychiatric disorders

**DOI:** 10.1186/2040-2392-5-1

**Published:** 2014-01-10

**Authors:** Holly N Cukier, Nicole D Dueker, Susan H Slifer, Joycelyn M Lee, Patrice L Whitehead, Eminisha Lalanne, Natalia Leyva, Ioanna Konidari, Ryan C Gentry, William F Hulme, Derek Van Booven, Vera Mayo, Natalia K Hofmann, Michael A Schmidt, Eden R Martin, Jonathan L Haines, Michael L Cuccaro, John R Gilbert, Margaret A Pericak-Vance

**Affiliations:** 1 John P. Hussman Institute for Human Genomics, University of Miami, Miller School of Medicine, 1501 NW 10th Avenue, BRB-314 (M860), Miami, FL, USA; 2 Dr. John T. Macdonald Foundation Department of Human Genetics, University of Miami, Miller School of Medicine, Miami, FL 33136, USA; 3 Center for Human Genetics Research, Vanderbilt University, Nashville, TN 37232-0700, USA

**Keywords:** Autism spectrum disorder (ASD), Identical by descent (IBD), Single nucleotide variant (SNV), Whole exome sequencing

## Abstract

**Background:**

Autism spectrum disorders (ASDs) comprise a range of neurodevelopmental conditions of varying severity, characterized by marked qualitative difficulties in social relatedness, communication, and behavior. Despite overwhelming evidence of high heritability, results from genetic studies to date show that ASD etiology is extremely heterogeneous and only a fraction of autism genes have been discovered.

**Methods:**

To help unravel this genetic complexity, we performed whole exome sequencing on 100 ASD individuals from 40 families with multiple distantly related affected individuals. All families contained a minimum of one pair of ASD cousins. Each individual was captured with the Agilent SureSelect Human All Exon kit, sequenced on the Illumina Hiseq 2000, and the resulting data processed and annotated with Burrows-Wheeler Aligner (BWA), Genome Analysis Toolkit (GATK), and SeattleSeq. Genotyping information on each family was utilized in order to determine genomic regions that were identical by descent (IBD). Variants identified by exome sequencing which occurred in IBD regions and present in all affected individuals within each family were then evaluated to determine which may potentially be disease related. Nucleotide alterations that were novel and rare (minor allele frequency, MAF, less than 0.05) and predicted to be detrimental, either by altering amino acids or splicing patterns, were prioritized.

**Results:**

We identified numerous potentially damaging, ASD associated risk variants in genes previously unrelated to autism. A subset of these genes has been implicated in other neurobehavioral disorders including depression (*SLIT3*), epilepsy (*CLCN2*, *PRICKLE1*), intellectual disability (*AP4M1*), schizophrenia (*WDR60*), and Tourette syndrome (*OFCC1*). Additional alterations were found in previously reported autism candidate genes, including three genes with alterations in multiple families (*CEP290*, *CSMD1*, *FAT1*, and *STXBP5*). Compiling a list of ASD candidate genes from the literature, we determined that variants occurred in ASD candidate genes 1.65 times more frequently than in random genes captured by exome sequencing (*P* = 8.55 × 10^-5^).

**Conclusions:**

By studying these unique pedigrees, we have identified novel DNA variations related to ASD, demonstrated that exome sequencing in extended families is a powerful tool for ASD candidate gene discovery, and provided further evidence of an underlying genetic component to a wide range of neurodevelopmental and neuropsychiatric diseases.

## Background

Autism spectrum disorders (ASDs) encompass a constellation of neurodevelopmental conditions characterized by three features: marked qualitative difficulties in social relatedness, communication, and behavior [1]. ASDs occur in approximately one of every 88 individuals and genetic studies to date demonstrate that the ASD etiology is highly complex with over 100 candidate genes being implicated in ASD etiology through linkage, association, and candidate gene studies [2,3]. This genetic complexity is compounded by the fact that many families present with private and rare alterations and that known pathogenic alterations can result in a variety of clinical consequences [4-6]. Indeed, genetic overlap has been reported between ASDs and other neurodevelopmental and neuropsychiatric disorders including attention deficit hyperactivity disorder (ADHD), intellectual disability, schizophrenia, and Tourette syndrome [7-11].

With the advent of whole exome sequencing, studies have been rapidly identifying rare genetic variants and pinpointing the causes of classic Mendelian disorders [12]. However, exome sequencing of more complex disorders, such as autism, have primarily focused either on simplex families to discover *de novo* alterations [13-17] or consanguineous families that carry recessive mutations [18,19]. In contrast, we designed a study to perform whole exome sequencing in extended, multiplex families with at least an affected cousin pair to identify potential new ASD loci. We hypothesized that identical by descent (IBD) filtering in these pedigrees would permit us to isolate genes contributing to ASD pathogenesis since these extended families are likely to carry novel ASD susceptibility loci of moderate to high effect. Our strategy discovered potentially damaging alterations in both known and novel ASD candidate genes, as well as in genes that carry variations known to be pathogenic in other neurological disorders.

## Methods

### Ethics statement

We ascertained individuals at the John P. Hussman Institute for Human Genomics (HIHG) at the University of Miami, Miller School of Medicine (Miami, FL, USA), the University of South Carolina (Columbia, SC, USA), and the Center for Human Genetics Research at Vanderbilt University (Nashville, TN, USA). Written informed consent was obtained from parents for all minor children and those who were unable to give consent. In addition, we obtained assent from all participants of the appropriate developmental and chronological age. All participants were ascertained using the protocol approved by the appropriate Institutional Review Boards. Patients were collected for this study for over a decade, with protocols and amendments being approved at each stage. Oversight of the study falls under the University of Miami (UM) Institutional Review Board (IRB). This study was approved by the UM Medical Sciences IRB Committee B members: Ofelia Alvarez MD, Abdul Mian PhD, Jose Castro MD, Rabbi Hector Epelbaum MA, Jean Jose DO, Howard Landy MD, Bruce Nolan MD FACP FAASM, Eric Zetka PharmD, and Liza Gordillo BA BS.

### Sample selection

One hundred and sixty-four individuals (100 ASD patients: 90 males and 10 females; 5 relatives with ASD features: 2 males and 3 females; and 59 unaffected relatives: 27 males and 32 females) from 40 ASD extended families were used in this study [Additional file 1: Table S1]. We define extended families as multiplex families with at least one pair of ASD affected cousins. Each family has between two to five ASD individuals and relationships to each other range from first degree relatives (that is, parent–child and siblings) to distant relatives (that is, third cousins). Thirty-nine families were of European ancestry, while a single family (7606) was of African ancestry. All participants were enrolled using protocols approved by the appropriate Institutional Review Boards. Core inclusion criteria for ASD individuals included: (1) between 3 and 21 years of age, (2) a presumptive clinical diagnosis of ASD, (3) an expert clinical determination of an ASD diagnosis using DSM-IV criteria [1] supported by the Autism Diagnostic Interview-Revised (ADI-R) [20], and (4) an IQ equivalent >35 or developmental level >18 months as determined by the Vineland Adaptive Behavior Scale (VABS) [21]. Diagnostic determination was based on review by a panel consisting of experienced clinical psychologists and a pediatric medical geneticist. In those instances where an ADI-R was not available, a best-estimate diagnosis was assigned using all available clinical information including clinician summaries, a caregiver report, and medical records. IQ was obtained for the majority of individuals from administration of any of several measures (for example, age appropriate Wechsler scale, Leiter intelligence test, or Mullen Scales of Early Learning, MSEL) or from medical records. A summary of the sample is provided in Additional file 1: Table S2. DNA was isolated either from saliva (n = 2) or whole blood collected via venipuncture (n = 162).

### Whole exome sequencing and variant detection

**Figure 1:**
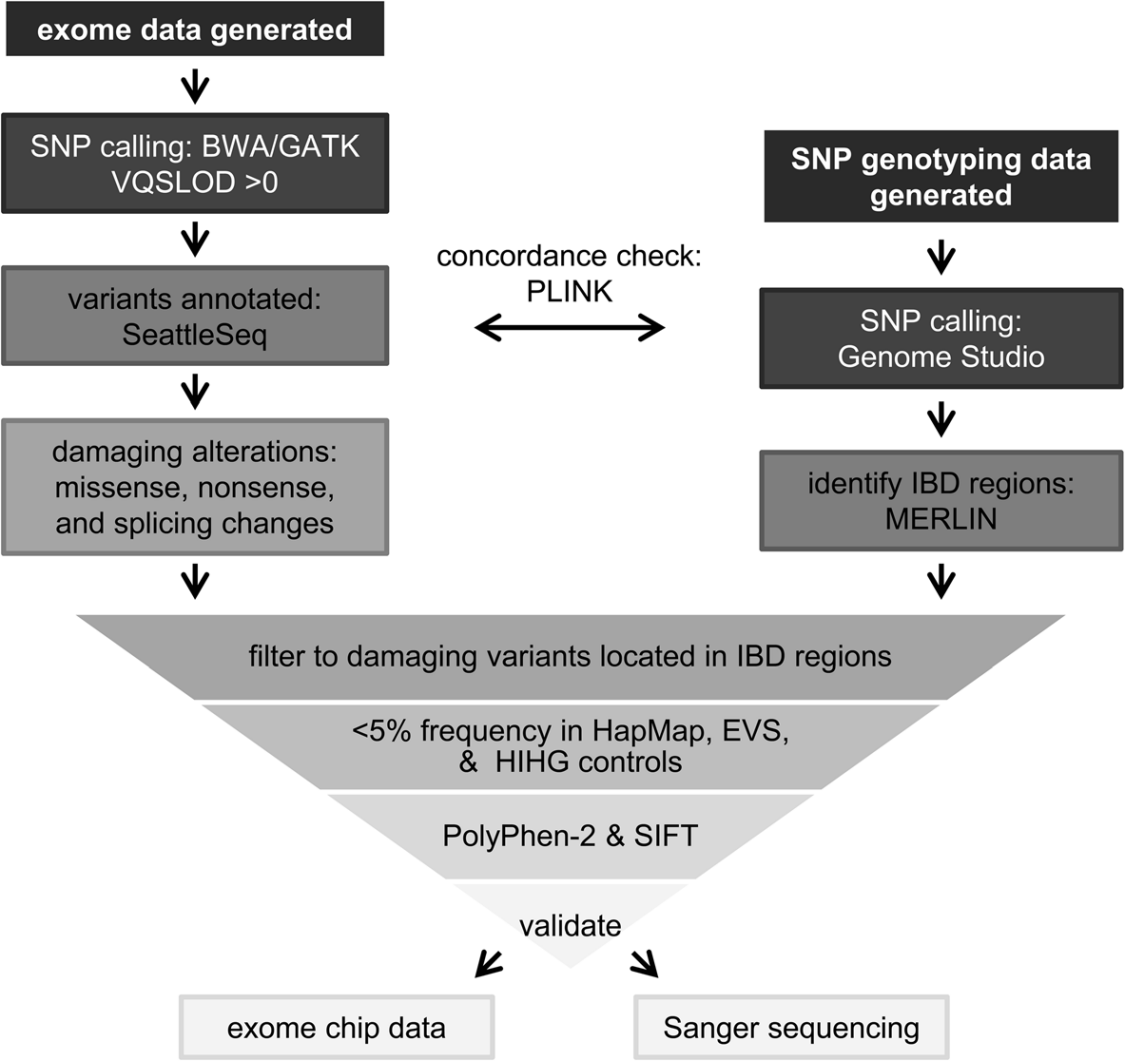
**Flowchart of sequencing and filtering methods to identify and prioritize IBD variants.** The data from whole exome sequencing as well as two genotyping platforms, whole genome SNP array and exome chip array, were each independently generated and processed. SNP array genotyping calls were compared to variants identified by exome sequencing as a quality check. Independent confirmation of calls from exome sequencing were made either by genotyping on the HumanExome BeadChip or by traditional Sanger sequencing. IBD, identical by descent.

One hundred and sixty-four samples from extended ASD families were prepared following standard Agilent (Santa Clara, CA, USA) and Illumina (San Diego, CA, USA) protocols for whole exome sequencing (Figure 1). Briefly, 3 μg of genomic DNA was sheared to approximately 150 to 200 base pair fragments with the Covaris (Woburn, MA, USA) E210 and sequence capture performed with Agilent’s SureSelect Human All Exon kit. Samples were hybridized for 24 hours. The initial 19 samples were prepared with the 38 Mb kit and run on Illumina’s Genome Analyzer IIx with each individual being run in two lanes. The remaining 145 samples were captured using the 50 Mb kit, indexed, and multiplexed to run three per lane on the Illumina Hiseq 2000. Paired end 2 × 100 sequencing was performed. Sequencing data was processed using the Illumina Real Time Analysis (RTA) base calling pipeline, initially with version 1.7 and with a subset being run on version 1.8. Alignment to the hg19 human reference genome was executed with the Burrows-Wheeler Aligner (BWA) and variant calling performed with the Genome Analysis Toolkit (GATK) [22,23]. GATK parameters included base quality score recalibration and duplicate removal [24]. Samples captured by the 38 Mb kit had an average depth of 64.4x, while the remaining samples processed with the 50 Mb kit had an average depth of 55.78x (Table 1). Variants were called at positions with a VQSLOD score greater than zero and minimum read depth of 4x. Alterations were annotated utilizing the SeattleSeq [25], PolyPhen-2 [26], and Sorting Intolerant From Tolerant (SIFT) programs [27]. The SeattleSeq program categorizes the two nucleotides flanking each exon as positions which, when altered, could potentially result in splicing alterations. In addition to samples from the autism extended families, 308 unrelated individuals of European ancestry negative for autism were internally processed at the HIHG. These HIHG control samples were captured with the Agilent SureSelect Human All Exon 50 Mb kit and processed according to the pipeline described above. Figure 1 outlines the steps used to generate and filter the data to the variants of interest. Genome wide SNP genotyping data on 159 samples was utilized to perform a quality check and confirm sample identity [28,29]. All but two samples passed quality control metrics. Therefore, we compared the single nucleotide variant (SNV) calls between the whole exome sequencing and SNP genotyping in 157 samples to confirm sample identity and found an average concordance of 98.3%.

**Table 1:** Average sequencing coverage and depth

		**On target coverage**			
**SureSelect kit**	**Percent on target**	**1x**	**8x**	**20x**	**Average depth**
38 Mb	63.34%	97.58%	81.68%	62.89%	64.41x
50 Mb	74.43%	95.24%	82.32%	65.37%	55.78x

### Genotyping and identity by descent filtering

Of the 164 samples selected for exome sequencing, 159 were also evaluated on one of four Illumina whole genome genotyping arrays: the Human 1Mv1 BeadChip (n = 129), the 1 M-DuoV3 BeadChip (n = 24), the HumanOmniExpress-12 v1.0 BeadChip (n = 4), or the HumanOmni2.5-4v1 BeadChip (n = 2). The 1 M and 1 M-Duo BeadChips were analyzed as previously described [28,29]. Samples processed on the OmniExpress BeadChip were prepared following Illumina’s Infinium HD Assay Ultra protocol, while those processed on the HumanOmni2.5 BeadChip followed Illumina’s Infinium HD Assay Super protocol. All chips were processed with automation on the Tecan (Männedorf, Switzerland) EVO-1 and BeadChips were scanned by either the Illumina BeadArray Reader or iScan. Data was extracted by the Genome Studio software from data files created by the iScan (Illumina) and a GenCall cutoff score of 0.15 was used. Samples on each of the four types of BeadChips were required to have a genotyping call rate of 98% or higher to pass quality control. Concordance between the genotypes of the variants identified through exome sequencing and genotyping was evaluated using the PLINK program [30]. All but two samples passed quality control metrics. Therefore, we compared the SNV calls between the whole exome sequencing and SNP genotyping in 157 samples to confirm sample identity and found an average concordance of 98.3%.

Genotyping information was further used to delineate IBD regions within each extended family. Only the 100 individuals with a confirmed ASD diagnosis were used to determine each family’s IBD regions. PLINK was employed for linkage disequilibrium (LD) pruning using the CEPH (Centre d'Etude du Polymorphisme Humain) CEU HapMap data for all families except 7606, for which the Yoruban (YRI) HapMap dataset was used [30]. The indep-pairwise option was utilized with a window size of 50, a step of 5, and an r^2^ threshold of 0.5. Next, these locations and their HapMap allele frequencies were analyzed in our dataset using the extended option in the MERLIN program using the 164 samples that were exome sequenced and 222 additional relatives [31]. To determine the start and stop positions of IBD sharing regions within each family, the MERLIN output was evaluated in a sliding window of ten SNVs, defining IBD as sharing at each location with a threshold >50%. Only regions shared across all available ASD individuals within a family were used to determine the IBD sharing segments.

To identify alterations inherited by all ASD individuals in a family from a single ancestor, whole exome sequencing data was restricted to IBD regions. Priority was also given to novel variants and rare SNVs. We define rare SNVs as those with a minor allele frequency (MAF), less than 5% in each of the three HapMap populations (African, Asian, or European), as well as in the 5,379 samples from the NHLBI Exome Sequencing Project, Exome Variant Server (EVS, version 5400) [32], and 308 HIHG control exomes. Novel variants were defined as those absent from the EVS, the 1000 Genomes Project [33], and dbSNP 134 [34]. Variants were evaluated for conservation with the Genomic Evolutionary Rate Profiling (GERP) score [35] and alterations measured for likelihood of having a damaging consequence on protein function through the PolyPhen-2 [27] and SIFT programs [28]. We also examined our results for overlap in genes previously reported in the literature and publically available databases (that is, SFARI Gene) to be associated with ASDs and other neurological disorders including ADHD, bipolar disorder, developmental delay, epilepsy, intellectual disability, major depression, obsessive compulsive disorder, schizophrenia, speech disorders, and Tourette syndrome [2,36,37].

### Enrichment of ASD genes

To determine whether there was enrichment of ASD genes in the IBD, damaging variants that were identified, results were compared to a list of 1,075 genes that included known and suspected ASD candidate genes [Additional file 1: Table S3]. This list was compiled from the review by Betancur [2], the ASD and candidate genes lists generated by Pinto and colleagues [37], three autism exome *de novo* papers [15-17], and the SFARI Gene database [36]. The *P* value was calculated using a hypergeometric distribution.

### Validation of variants

Of the 164 individuals from ASD extended families included in this study, 100 samples were also run on the Infinium HumanExome 12v1 BeadChip (Illumina). Exome chips were prepared following the manufacturer’s Infinium HD Assay Ultra protocol and automated using the Tecan EVO-1, as described above. Samples were required to have a genotyping call rate of 98% or higher to pass quality control. Variant calls were compared between the exome sequencing and genotyping with the PLINK program [30]. Between the exome sequencing and exome genotyping platforms, 446 changes were concordant, while only one variant was found to be discordant. A subset of variants was also validated by Sanger sequencing. Variants present in multiple families were prioritized, as were those occurring in genes with previous evidence implicating them in neurodevelopmental and neuropsychiatric disorders. Primers were created using the Primer3 v0.4.0 program (http://fokker.wi.mit.edu/primer3/input.htm) and the UCSC reference genome (GRCh37/hg19). Sequencing reactions were performed with the Big Dye Terminator v3.1, run on an Applied Biosystems 3730xl DNA Analyzer (Life Technologies, Carlsbad, CA, USA), and evaluated in the Sequencher v4.10.1 program (Gene Codes Corporation, Ann Arbor, MI, USA). Fifty-seven IBD changes were validated via Sanger sequencing, while one position failed to validate. With a total of 503/505 SNVs (99.6%) being concordant in two independent platforms, we determined that there is a relatively low false positive rate of variant calling in the filtered exome sequencing data.

## Results and discussion

### Identification and validation of rare and potentially damaging variants

Whole exome sequencing was performed in 164 individuals from 40 families to detect potentially causative variants [Additional file 1: Table S1 and S2]. SNP genotyping data on these 164 individuals and 222 relatives was used to isolate genomic areas inherited from a common ancestor, or IBD, and shared between ASD relatives (Figure 1). Following variant calling of the exome data and rigorous quality control (see Methods section), each family had SNVs at approximately 90,000 unique locations. We tested heterozygous, homozygous, and X-linked inheritance models using the same scheme (Figure 1). To investigate the first model, variants were filtered to include only IBD, heterozygous alterations present in all affected individuals within a family and predicted to be detrimental by either altering amino acids or splicing patterns. Variants were further parsed to include only those that were novel or relatively rare (MAF, <5%) in HapMap populations, EVS exomes, and 308 internally processed HIHG control exomes. A total of 742 IBD, heterozygous alterations in 690 genes were identified across the 40 families. This method was repeated with a homozygous model but no alterations survived the filtering process. Sixteen of the 40 families conformed to a possible X-linked pattern of inheritance and variants in three additional genes were found [Additional file 1: Table S1]. Therefore, a total of 745 rare, predicted damaging, alterations in 693 genes were identified. Three families did not demonstrate IBD co-segregation of any SNVs, while the remaining 37 families had at least two segregating SNVs. We then validated 502 of the 745 IBD variants of interest by one of two methods: Sanger sequencing or SNP genotyping [Additional file 1: Table S4].

### Genes identified with more than one variant

**Figure 2:**
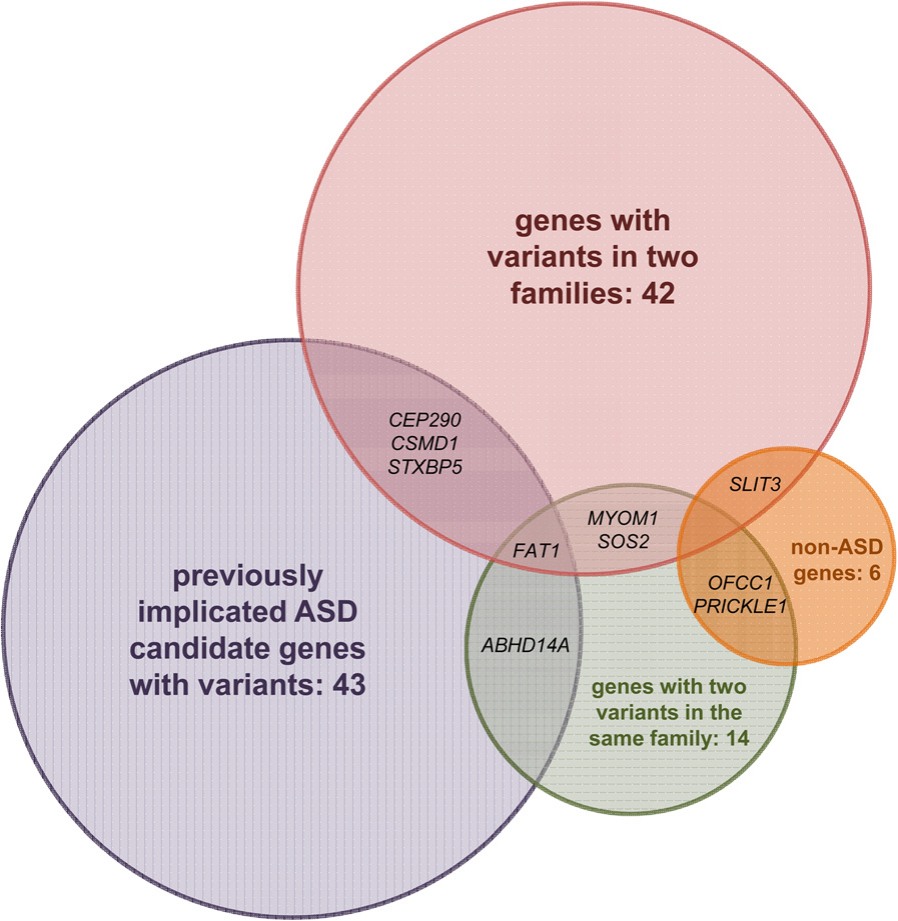
**Diagram of genes of high interest.** The area of each circle corresponds to the number of genes identified in one of four categories: genes previously implicated as ASD candidates, non-ASD genes that have been implicated in other neurodevelopmental and neuropsychiatric disorders, genes found to have damaging variants in more than one family, and genes that carry two damaging alterations in the same family. ASD, autism spectrum disorder.

We identified 36 genes that had segregating SNVs in at least two families (Figure 2, Additional file 1: Table S5). To put these new findings in context, we examined the ASD pedigrees for the presence of additional neurobehavioral features in both affected individuals and obligate carriers of the mutations of interest. While 32 of these genes have not been previously linked to ASDs, one of them, *SLIT3*, has been associated with another neuropsychiatric disorder (Table 2). Duplications overlapping the *SLIT3* gene were previously found to be overrepresented in individuals with major depressive disorder [38]. In our study, the two families carrying an alteration in *SLIT3*, 17342 and 18074, also presented with a history of depression in three of the four obligate carriers (all female); the fourth obligate carrier was male and had no reported neuropsychiatric traits. In addition, four ASD candidate genes were found with alterations in more than one extended family: *CEP290*, *CSMD1*, *FAT1*, and *STXPB5*. Alterations in *CEP290* have been connected to ASDs and intellectual disability and were previously identified in patients diagnosed with a wide variety of ciliopathies including Bardet-Biedl, Joubert, Meckel-Gruber, and Senior-Løken syndromes (http://medgen.ugent.be/cep290base/) [39]. Along with being linked to ASDs, *CSMD1* has been associated with schizophrenia and is a known target of mir-137, a microRNA that regulates neuronal maturation and adult neurogenesis [40-43]. *FAT1* is a candidate for both ASD and bipolar disorder and is a member of the cadherin gene family [15,44]. While we did not identify individuals with mutations in *FAT1* who presented with bipolar disorder, the two obligate carriers (female) in family 17545 reported a history of major depression. Lastly, a deletion across *STXBP5* was previously reported in a patient with autism, intellectual disabilities, and seizures [45]. *STXBP5* functions in neuronal guidance and synaptic transmission [46]. In our first family identified with a *STXBP5* variant, 37994, we observed intellectual disability in both of the ASD individuals as well as seizures in the proband (1). In addition, the mother (1001) of the proband was reported to have bipolar disorder. In the second family carrying a mutation in *STXBP5*, 7623, we observed seizures in one of the affected individuals (101) as well as migraines in his mother (1007), an obligate carrier. These results augment the growing evidence supporting a genetic overlap between a wide variety of neurodevelopmental and neuropsychiatric disorders [7-11].

**Table 2:** Damaging, validated variants in genes previously implicated in ASD or other disorders in multiple families

**Gene**	**Family**	**Position (Hg19)**	**dbSNP**	**Nucleotide**	**Amino acid**	**HIHG control chromosomes**	**Disorder**	**References**
*CEP290*	37425	chr12:88472996	rs61941020	C > T	Arg1746Gln	3/610	ASD, ID	Coppieters *et al*., 2010 [39]
	37117	chr12:88508258	rs79705698	T > C	Asp664Gly	15/612	ASD, ID	Coppieters *et al*., 2010 [39]
*CSMD1*	37117	chr8:2965294	-	G > C	Pro2262Ala	2/614	ASD, SZ	Havik *et al*., [40]
	17122	chr8:3253832	-	C > T	Gly827Asp	0/604	ASD, SZ	Havik *et al*., [40]
*FAT1*	37037	chr4:187518041	rs72716244	T > C	Asp4218Gly	11/610	ASD, BD	Blair *et al*., [43], Neale *et al*., [15]
	17545	chr4:187549364	rs111886222	G > A	Thr1585Met	5/614	ASD, BD	Blair *et al*., [43], Neale *et al*., [15]
	17545	chr4:187557908	rs113970444	C > T	Arg1268Gln	5/608	ASD, BD	Blair *et al*., [43], Neale *et al*., [15]
*SLIT3*	17342, 18074	chr5:168180047	rs34260167	C > T	Ser629Asn	7/616	MD	Glessner *et al*., [38]
*STXBP5*	37994	chr6:147635108	rs144099092	A > G	Leu412Val	2/614	ASD	Davis *et al*., [44]
	7623	chr6:147636753	rs148830578	T > A	Try502Cys	2/614	ASD	Davis *et al*., [44]

ASD, autism spectrum disorder; BD, bipolar disorder; HIHG, Hussman Institute for Human Genomics; ID, intellectual disability; MD, major depression; SZ, schizophrenia.

Fourteen genes segregated two heterozygous and damaging SNVs in cis within a single family; 12 of these genes have no prior evidence of a connection to ASDs (Figure 2, Additional file 1: Table S6). Two novel genes have been identified in other neurodevelopmental and neuropsychiatric disorders: *PRICKLE1* and *OFCC1* (Table 3). Family 37232 carries two changes in *PRICKLE1*, a gene involved in neurite outgrowth which has been linked to epilepsy and neural tube defects [47-50]. Interestingly, the proband of this family (1) was reported to have a history of seizures. Independently, Paemka and colleagues implicated *PRICKLE1* as an ASD gene through extensive *in vivo* and *in vitro* functional analysis [51], thereby demonstrating the power of the extended family approach to identify novel ASD candidates. Moreover, *OFCC1*, a gene linked with Tourette syndrome [52], was found to have both a missense and a nonsense alteration in family 7606, the one family of African ancestry in this study. In this family, we observed self-injurious behaviors in all three affected individuals as well as seizures in one affected (2061). In addition, two ASD genes were found to carry variants in cis: *ABHD14A* and *FAT1*. Family 17351 carries two changes in *ABHD14A*, a gene involved in cerebellar development [53,54]. In this family, one affected individual (105) was described as having intellectual disability and a male obligate carrier (1000) was reported to have bipolar disorder. Another ASD candidate gene, *FAT1*, has two alterations in family 17545, as well as a distinct variant detected in family 37037 [15,44]. As mentioned above, family 17545 reported that both obligate carriers in the family have a history of major depression.

**Table 3:** Families with multiple damaging, validated variants in the same, previously implicated gene

**Gene**	**Family**	**Position (Hg19)**	**dbSNP**	**Nucleotide**	**Amino acid**	**HIHG control chromosomes**	**Disorder**	**References**
*ABHD14A*	17351	chr3:52011912	rs17849626	G > A	Arg32Gln	25/614	ASD	Casey *et al*., [53]
		chr3:52014897	rs61729088	C > G	Arg227Gly	2/616	ASD	Casey *et al*., [53]
*FAT1*	17545	chr4:187549364	rs111886222	G > A	Thr1584Met	5/614	ASD, BD	Blair *et al*., [43], Neale *et al*., [15]
		chr4:187557908	rs113970444	C > T	Arg1268Gln	5/608	ASD, BD	Blair *et al*., [43], Neale *et al*., [15]
*OFCC1*	7606	chr6:9809860	-	C > T	Arg538Gln	0/602	TS	Sundaram *et al*., [51]
		chr6:9900660	rs148761621	C > A	Glu204Stop	0/614	TS	Sundaram *et al*., [51]
*PRICKLE1*	37232	chr12:42862463	rs61924369	C > T	Glu185Lys	0/614	E	Bassuk *et al*., [47], Tao *et al*., [49]
		chr12:42864125	-	C > G	Val57Leu	0/614	E	Bassuk *et al*., [47], Tao *et al*., [49]

ASD, autism spectrum disorder; BD, bipolar disorder; E, epilepsy; HIHG, Hussman Institute for Human Genomics; TS, Tourette syndrome.

### Single alterations found in genes related to neurological disorders

Three additional genes related to other neuropsychiatric and neurodevelopmental disorders were each recognized to carry a single IBD alteration: *AP4M1* (intellectual disability [55]), *CLCN2* (epilepsy [56]), and *WDR60* (intellectual disability and schizophrenia [57], Additional file 1: Table S7). Moreover, an additional 38 known or suspected ASD genes were identified with one variant, including *AGAP1*[58], *CDH9*[28,59], *DLGAP2*[60], *FBXO40*[61], *GRIN3B*[13], *NRXN2*[62], and *SYNE2*[14]. Following an X-linked pattern of inheritance, hemizygous alterations were identified and validated in *SYN1*, a gene which encodes a synaptic vesicle phosphoprotein and has been previously connected to both autism and epilepsy [63-65]. Both ASD individuals carrying the *SYN1* alteration in family 37674 have a history of epilepsy. Interestingly, Paemka and colleagues independently show that SYN1 co-immunoprecipitated with PRICKLE1 in mouse brain and that the proteins co-localized in *Drosophila* neurons [51], demonstrating a conserved physical interaction between a newly identified candidate gene, *PRICKLE1*, and an established ASD gene, *SYN1*, and supporting the validity of our exome filtering method. This provides additional evidence that investigating genes and pathways that interact with known ASD candidates will likely prove a fruitful area for the identification of ASD related genes.

Further potentially novel ASD candidates identified by exome sequencing include genes with previous clinical and molecular evidence supporting a neuronal function such as *CIC*, *GLUD2*, *NTSR2*, *RODG1*, and *SEZ6. CIC* is a genetic modifier of the neurodegenerative disorder spinocerebellar ataxia [66]. *GLUD2* plays a role in postsynaptic density formation in the cerebellum and modifies the age of onset of Parkinson’s disease patients [67,68]. *NTSR2* is a G protein-coupled receptor for neurotensin that is widely expressed throughout the brain [69]. *RODG1* mutations result in Kohlschütter-Tönz syndrome, a disorder that presents with epilepsy and developmental delay, both features found in children with autism [70]. Lastly, *SEZ6* acts in dendritic arborization and has been associated with seizures in a Chinese cohort [71,72]. This category of genes, along with genes identified to carry alterations in more than one family, may be the most fruitful groups of novel ASD candidates for future investigations.

### IBD filtering enriches for ASD candidate genes

In order to determine whether our filtering method enriched for ASD specific variants, we generated a list of 1,075 known and suspected ASD candidates from the literature and the SFARI Gene database [2,15-17,36,37]. When this ASD candidate list was compared to the 693 genes carrying variants meeting the above filter criteria, we found that 5.7% of the genes in the ASD list met our criteria compared to only 3.4% of all genes captured by the exome sequencing; thus, we were 1.65 times more likely to find a variant in an ASD candidate gene than a random gene captured by exome sequencing (*P* = 8.55 × 10^-5^). We therefore concluded that there was a significant enrichment of ASD candidate genes carrying damaging, segregating variants, supporting our hypothesis that whole exome sequencing of extended autism families is a reliable approach to identify new autism genes.

## Conclusions

Our results have identified several new genes that likely play a role in ASD and provide additional support for the role of other proposed ASD genes. These data reinforce two emerging observations about ASDs. The first is the extreme level of genetic heterogeneity, with no locus contributing to more than 1% of ASD cases [2,73]. The second is that there is significant overlap between the genetic etiology of ASD and other neuropsychiatric and neurodevelopmental disorders [7-11]. These data, taken together with the increasing amount of functional data available for many of these genes such as *PRICKLE1*[51], highlight the enormous complexity of ASD and the difficulties in resolving this enigma.

## Abbreviations

ADHD: Attention deficit hyperactivity disorder; ADI-R: Autism diagnostic interview-revised; ASD: Autism spectrum disorder; BWA: Burrows-Wheeler Aligner; CEU: The CEPH (Centre d'Etude du Polymorphisme Humain) HapMap cohort; DSM-IV: Diagnostic and statistical manual of mental disorders fourth edition; EVS: Exome variant server; GATK: Genome analysis toolkit; GERP: Genomic evolutionary rate profiling; HIHG: Hussman institute for human genomics; IBD: Identical by descent; IQ: Intelligence quotient; IRB: Institutional review board; LD: Linkage disequilibrium; MAF: Minor allele frequency; MSEL: Mullen scales of early learning; RTA: Real time analysis; SIFT: Sorting intolerant from tolerant; SNP: Single nucleotide polymorphism; SNV: Single nucleotide variant; UCSC: University of California Santa Cruz; UM: University of Miami; VABS: Vineland adaptive behavior scale; YRI: Yoruban HapMap cohort.

## Competing interests

The authors declare that they have no competing interests.

## Authors’ contributions

HNC, JLH, MLC, JRG, and MAPV conceived and designed the experiments. HNC prepared samples for exome sequencing, analyzed exome sequencing data, and wrote the manuscript. JML and MLC collected the samples, made diagnoses, and interpreted the clinical data from the ASD families. PLW, EL, NL, IK, RCG, and WFH performed the exome sequencing and exome chip genotyping. SHS, DVB, MAS, ERM, and MAPV analyzed the exome sequencing data. NDD analyzed the exome chip data. VM and NKH performed the Sanger sequencing validation. NDD, ERM, JLH, MLC, JRG, and MAPV edited the manuscript. The authors jointly discussed the experimental results throughout the duration of the study. All authors read and approved the final manuscript.

## Supplementary Material

Additional file 1 **This file contains the seven tables listed below: Table S1 - Extended families structure. ** Table S2 - Clinical information on individuals with ASDs. Table S3 - ASD candidate genes. Table S4 - Variants identified in exome sequencing and validated by a second platform. Table S5 - Genes with damaging, validated variants in more than one family. Table S6 - Families with multiple damaging, validated variants in the same gene. Table S7 - Damaging, validated variants in genes previously implicated in ASD or other disorders. Click here for file
