# Supplementary material for: Exome sequencing of extended families with autism reveals genes shared across neurodevelopmental and neuropsychiatric disorders
Source: Mol Autism. 2014 Jan 10;5:1. doi: 10.1186/2040-2392-5-1 (PMC3896704; doi:10.1186/2040-2392-5-1)
Supplement: Additional file 1 — This file contains the seven tables listed below: Table S1 - Extended families structure. Table S2 - Clinical information on individuals with ASDs. Table S3 - ASD candidate genes. Table S4 - Variants identified in exome sequencing and validated by a second platform. Table S5 - Genes with damaging, validated variants in more than one family. Table S6 - Families with multiple damaging, validated variants in the same gene. Table S7 - Damaging, validated variants in genes previously implicated in ASD or other disorders. [file 2040-2392-5-1-S1.doc]

| **Supporting Table 1 - Extended Families Structure** | | | | | | |
| --- | --- | --- | --- | --- | --- | --- |
|  |  |  |  |  |  |  |
|  | **ASD Individuals Sequenced** | |  | **Obligate Carriers** | |  |
| **Family^+^** | **Female** | **Male** | **Relationships between Individuals with ASDs** | **Female** | **Male** | **Potential X-linked Inheritance** |
| 3836 | 0 | 2 | 1st cousins | 1 | 1 | no |
| 7435* | 0 | 2 | 2nd cousins | 4 | 0 | yes |
| 7503 | 0 | 2 | 2nd cousins once removed | 3 | 2 | no |
| 7506^ | 1 | 3 | 1st and 2nd cousins, siblings | 0 | 3 | no |
| 7531^ | 1 | 2 | 1st cousins, parent | 1 | 1 | no |
| 7590 | 0 | 2 | 1st cousins, consanguineous family | 0 | 2 | no |
| 7606 | 1 | 2 | 1st cousins twice removed, siblings | 4 | 1 | yes |
| 7623 | 0 | 3 | 2nd cousins, siblings | 3 | 1 | yes |
| 7637^ | 0 | 4 | 1st cousins, parent, siblings | 0 | 2 | no |
| 7658 | 0 | 2 | 2nd cousins | 0 | 4 | no |
| 7663 | 1 | 3 | 1st cousins, full and half siblings | 2 | 0 | yes |
| 7713 | 0 | 3 | 1st cousins, siblings | 1 | 1 | no |
| 7728 | 0 | 2 | 2nd cousins | 1 | 3 | no |
| 7745 | 0 | 2 | 2nd cousins | 3 | 1 | yes |
| 7784 | 1 | 2 | 1st cousins, siblings | 1 | 1 | no |
| 7797 | 0 | 2 | 2nd cousins | 3 | 1 | no |
| 7870 | 1 | 1 | 2nd cousins | 3 | 1 | yes |
| 7872* | 0 | 3 | 1st and 2nd cousins | 3 | 2 | no |
| 7936 | 1 | 1 | 1st cousins | 1 | 1 | yes |
| 17122 | 0 | 3 | 1st cousins, siblings | 2 | 0 | yes |
| 17142 | 0 | 3 | 2nd cousins, siblings | 3 | 1 | no |
| 17245 | 0 | 4 | 2nd cousins once removed, siblings | 2 | 3 | no |
| 17302^ | 1 | 3 | 1st cousins, siblings | 1 | 1 | no |
| 17342 | 0 | 2 | 1st cousins | 2 | 0 | yes |
| 17351 | 0 | 2 | 1st cousins | 0 | 2 | no |
| 17478 | 1 | 2 | 1st cousins | 1 | 2 | no |
| 17545 | 0 | 2 | 1st cousins | 2 | 0 | yes |
| 17678 | 0 | 2 | 1st cousins | 1 | 1 | no |
| 18074 | 0 | 2 | 1st cousins | 1 | 1 | no |
| 37024 | 0 | 2 | 2nd cousins | 2 | 2 | no |
| 37037 | 0 | 2 | 1st cousins | 1 | 1 | no |
| 37117 | 0 | 2 | 1st cousins once removed | 4 | 0 | yes |
| 37150 | 0 | 2 | 1st cousins | 2 | 0 | yes |
| 37232 | 0 | 2 | 1st cousins | 1 | 1 | no |
| 37309* | 1 | 2 | 3rd cousins, siblings | 5 | 1 | yes |
| 37425 | 0 | 2 | 1st cousins | 2 | 0 | yes |
| 37534* | 0 | 2 | 3rd cousins | 3 | 1 | no |
| 37674 | 0 | 2 | 1st cousins | 2 | 0 | yes |
| 37994* | 0 | 2 | 1st cousins | 2 | 0 | yes |
| 39312 | 0 | 2 | 2nd cousins | 1 | 3 | no |
| Total: | 10 | 90 |  | 74 | 48 |  |
| ^+^affected individuals in each family were captured with the 50 Mb kit unless otherwise annotated | | | | | | |
| *all affected individuals in this family were captured with the 38 Mb kit | | | | | | |
| ^affected individuals in this family were captured either with the 38 Mb kit or the 50 Mb kit | | | | | | |

| **Supporting Table 2 - Clinical Information on Individuals with ASDs** | | | | | | | | | |
| --- | --- | --- | --- | --- | --- | --- | --- | --- | --- |
|  |  |  |  |  |  |  |  |  |  |
| **Family** | **Sample** | **Sex** | **Age of Recognition'^◼^** | **ADI Age'** | **Single Words'^◆^** | **First Walked'^◆^** | **Adap Behavior Standard'** | **IQ Test^#** | **IQ^** |
| 3836 | 1 | M | 12 | 68 | 13 | 10 | 84 | MSEL | 51 |
| 3836 | 101 | M | 12 | 51 | 993 | 15 | 66 |  |  |
| 7435 | 1 | M | 2 | 343 | 12 | 21 | 19 | LIT | 54 |
| 7435 | 112 | M |  |  |  |  | 20 |  |  |
| 7503 | 1 | M | 7 | 97 | 994 | 13 | 22 |  |  |
| 7503 | 1021 | M | 992 | 413 | 102 | 999 |  |  |  |
| 7506 | 1 | F | 992 | 120 | 36 | 9 | 101 | LIT | 123 |
| 7506 | 120 | M | 24 | 61 | 36 | 10 | 73 |  |  |
| 7506 | 9006 | M | 84 | 201 | 9 | 11 |  | WISC IV | 102 |
| 7506 | 9009 | M | 30 | 105 | 36 | 12 |  | LIT | 98 |
| 7531 | 100 | M | 18 | 97 | 24 | 15 | 62 | WAIS III | 90 |
| 7531 | 113 | M | 30 | 145 | 15 | 13 |  |  |  |
| 7531 | 1001 | F |  |  |  |  |  |  |  |
| 7590 | 1 | M | 7 | 92 | 994 | 999 | 23 |  |  |
| 7590 | 101 | M | 12 | 30 | 994 | 15 | 31 |  |  |
| 7606 | 1 | M | 24 | 92 | 18 | 12 | 25 |  |  |
| 7606 | 100 | F | 36 | 75 | 18 | 10 | 58 |  |  |
| 7606 | 2061 | M | 42 | 209 | 993 | 11 |  |  |  |
| 7623 | 1 | M | 992 | 140 | 24 | 20 | 71 | WISC-R/III | 61 |
| 7623 | 100 | M | 48 | 113 | 24 | 999 | 88 | LIT | 89 |
| 7623 | 101 | M | 12 | 127 | 36 | 19 | 24 |  |  |
| 7637 | 1 | M | 21 | 124 | 15 | 996 | 73 | LIT | 50 |
| 7637 | 103 | M | 17 | 58 | 46 | 14 | 43 | LIT | 91 |
| 7637 | 104 | M | 12 | 65 | 48 | 17 | 111 |  |  |
| 7637 | 1002 | M |  |  |  |  |  |  |  |
| 7658 | 1 | M | 1 | 58 | 12 | 15 | 64 | LIT | 146 |
| 7658 | 101 | M | 992 | 192 | 30 | 14 | 24 |  |  |
| 7663 | 1 | M | 999 | 63 | 999 | 999 | 44 | MSCA | 51 |
| 7663 | 100 | F | 2 | 54 | 997 | 19 | 55 | LIT | 30 |
| 7663 | 132 | M | 992 | 328 | 36 | 13 |  | WISC-R/III | 121* |
| 7663 | 134 | M | 30 | 151 | 12 | 13 |  |  |  |
| 7713 | 1 | M | 27 | 60 | 40 | 12 | 62 |  |  |
| 7713 | 100 | M | 20 | 45 | 12 | 11 | 51 |  |  |
| 7713 | 101 | M |  |  |  |  |  |  |  |
| 7728 | 1 | M | 17 | 110 | 12 | 10 |  | LIT | 57 |
| 7728 | 111 | M | 13 | 87 | 36 | 14 |  |  |  |
| 7745 | 1 | M | 21 | 204 | 45 | 12 | 52 |  |  |
| 7745 | 104 | M | 15 | 139 | 48 | 14 | 83 |  |  |
| 7784 | 1 | F | 20 | 63 | 17 | 13 | 78 | WISC IV | 104 |
| 7784 | 100 | M | 13 | 40 | 13 | 13 | 76 | LIT | 88 |
| 7784 | 106 | M |  |  |  |  |  |  |  |
| 7797 | 1 | M | 16 | 126 | 57 | 9 |  | WISC-R/III | 90** |
| 7797 | 106 | M | 42 | 175 | 36 | 15 |  |  |  |
| 7870 | 1 | M | 24 | 58 | 14 | 11 | 58 |  |  |
| 7870 | 106 | F | 18 | 134 | 996 | 12 | 103 |  |  |
| 7872 | 1 | M | 24 | 110 | 11 | 7 | 82 | CELF-III | 73 |
| 7872 | 103 | M |  |  |  |  |  |  |  |
| 7872 | 104 | M | 24 | 178 | 13 | 10 | 65 | LIT | 87 |
| 7936 | 1 | F | 992 | 131 | 14 | 15 | 61 |  |  |
| 7936 | 110 | M | 18 | 116 | 996 | 10 | 54 |  |  |
| 17122 | 1 | M | 20 | 148 | 24 | 18 | 61 | WAIS III | 46 |
| 17122 | 100 | M | 11 | 182 | 48 | 18 | 55 | WISC IV | 46 |
| 17122 | 101 | M | 96 | 192 | 12 | 11 | 65 | WISC-R/III | 99 |
| 17142 | 1 | M | 3 | 49 | 33 | 17 | 71 | WISC IV | 98 |
| 17142 | 101 | M | 12 | 64 | 51 | 18 | 67 |  |  |
| 17142 | 106 | M | 36 | 242 | 18 | 9 |  |  |  |
| 17245 | 1 | M | 24 | 191 | 13 | 14 | 78 | WISC-R/III | 97 |
| 17245 | 9006 | M | 57 | 108 | 7 | 11 |  |  |  |
| 17245 | 9007 | M |  |  |  |  |  |  |  |
| 17245 | 9008 | M |  |  |  |  |  |  |  |
| 17302 | 1 | M | 24 | 92 | 33 | 12 | 54 | WISC IV | 70 |
| 17302 | 100 | M | 12 | 38 | 24 | 12 | 67 | LIT | 90 |
| 17302 | 112 | M | 16 | 44 | 14 | 10 | 66 |  |  |
| 17302 | 113 | F | 14 | 89 | 11 | 13 | 73 |  |  |
| 17342 | 1 | M | 6 | 193 | 24 | 996 |  |  |  |
| 17342 | 100 | M | 14 | 175 | 12 | 9 |  | WISC-R/III | 112 |
| 17351 | 1 | M | 14 | 218 | 42 | 12 |  | WAIS IV | 68 |
| 17351 | 105 | M | 15 | 147 | 996 | 9 | 20 |  |  |
| 17478 | 1 | M | 30 | 111 | 996 | 14 | 94 | CELF | 75 |
| 17478 | 103 | M | 24 | 244 | 42 | 12 |  | WISC-R/III | 92 |
| 17478 | 104 | F | 42 | 212 | 24 | 13 |  | WISC-R/III | 106 |
| 17545 | 1 | M |  |  |  |  | 31 | WISC-R/III | 50 |
| 17545 | 100 | M |  |  |  |  | 45 | WISC-R/III | 64 |
| 17678 | 1 | M | 18 | 135 | 996 | 11 | 80 |  |  |
| 17678 | 107 | M | 21 | 166 | 11 | 11 | 82 |  |  |
| 18074 | 1 | M | 12 | 83 | 12 | 17 |  |  |  |
| 18074 | 109 | M | 24 | 117 | 993 | 11 | 59 |  |  |
| 37024 | 1 | M |  |  |  |  |  |  |  |
| 37024 | 102 | M | 13 | 130 | 84 | 14 |  |  |  |
| 37037 | 1 | M | 30 | 86 | 13 | 11 | 100 | WISC IV | 97 |
| 37037 | 110 | M | 24 | 138 | 13 | 15 |  | WPPSI-R | 79 |
| 37117 | 1 | M |  |  |  |  | 90 | WISC IV | 106 |
| 37117 | 1003 | M | 36 | 243 | 36 | 10 | 74 | WISC-R/III | 75 |
| 37150 | 1 | M | 36 | 270 | 996 | 9 | 69 | WISC-R/III | 81 |
| 37232 | 1 | M | 12 | 70 | 33 | 11 | 101 | WPPSI III* | 104* |
| 37232 | 108 | M | 60 | 138 | 14 | 12 | 87 |  |  |
| 37309 | 1 | M | 12 | 89 | 36 | 14 | 62 |  |  |
| 37309 | 100 | F | 18 | 102 | 24 | 16 | 99 |  |  |
| 37309 | 101 | M |  |  |  |  | 56 |  |  |
| 37425 | 1 | M | 36 | 192 | 18 | 10 | 80 | WISC IV** | 25** |
| 37425 | 100 | M | 48 | 156 | 12 | 12 | 79 |  |  |
| 37534 | 1 | M | 24 | 100 | 15 | 9 | 107 |  |  |
| 37534 | 104 | M | 21 | 182 | 51 | 11 |  |  |  |
| 37674 | 1 | M | 42 | 144 | 24 | 14 | 70 | WISC IV | 116 |
| 37674 | 100 | M | 13 | 274 | 48 | 11 | 93 | WAIS III | 103 |
| 37994 | 1 | M | 8 | 188 | 24 | 14 | 61 |  |  |
| 37994 | 103 | M | 28 | 297 | 18 | 11 |  | WAIS III | 50 |
| 39312 | 1 | M |  |  |  |  | 93 | WISC IV | 112 |
| 39312 | 105 | M | 9 | 62 | 36 | 13 | 63 |  |  |
| 37150 | 106 | M |  |  |  |  |  |  |  |
| ‘Age is displayed in months | | | |  |  |  |  |  |  |
| ^◼^992 - parents have been worried since birth, 999 - not known or not asked | | | | | | | |  |  |
| ^◆^993 - had some words, then lost, 994 - milestone not reached, 996 - not known, but apparently normal, 997 - not known, but apparently delayed, 999 - not known or not asked | | | | | | | | | |
| ^Denotes full IQ score unless otherwise specified, *verbal IQ score, **nonverbal IQ score | | | | | | | |  |  |
| ^#^CELP - Clinical Evaluation of Language Fundamentals, LIT - Leiter Intelligence Test, MCSA - Merrill-Palmer Scales of Mental Development, MSEL - Mullen Scales of Early Learning, WAIS - Wechsler Adult Intelligence Scale, WISC - Wechsler Intelligence Scale for Children, WPPSI - Wechsler Preschool and Primary Scale of Intelligence | | | | | | | | | |

| **Supporting Table 3 - ASD Candidate Genes** | |
| --- | --- |
|  |  |
| **Gene** | **Reference** |
| *A2BP1* | Pinto et al., 2010 |
| *A2M* | Sanders et al., 2012 |
| *AADACL2* | Sanders et al., 2012 |
| *ABAT* | SFARI database |
| *ABCA12* | Neale et al., 2012 |
| *ABCA13* | Neale et al., 2012 |
| *ABCA2* | O'Roak et al., 2012 |
| *ABCC12* | Neale et al., 2012 |
| *ABI3BP* | Sanders et al., 2012 |
| *ACLY* | O'Roak et al., 2012 |
| *ACP2* | O'Roak et al., 2012 |
| *ACSL4 (FACL4)* | Betancur et al., 2011 |
| *ADA* | SFARI database |
| *ADAM22* | Neale et al., 2012 |
| *ADAM33* | Sanders et al., 2012 |
| *ADARB1* | SFARI database |
| *ADCY5* | O'Roak et al., 2012, SFARI database |
| *ADK* | SFARI database |
| *ADNP* | O'Roak et al., 2012, SFARI database |
| *ADORA2A* | SFARI database |
| *ADRB2* | SFARI database |
| *ADSL* | Betancur et al., 2011, Pinto et al., 2010, SFARI database |
| *AFF2* | Betancur et al., 2011, Pinto et al., 2010, SFARI database |
| *AFF4* | Sanders et al., 2012, SFARI database |
| *AGAP1* | Pinto et al., 2010 |
| *AGBL4* | SFARI database |
| *AGK* | Sanders et al., 2012 |
| *AGMO* | SFARI database |
| *AGTR2* | Betancur et al., 2011, Pinto et al., 2010, SFARI database |
| *AHI1* | Betancur et al., 2011, Pinto et al., 2010, SFARI database |
| *AHNAK2* | Neale et al., 2012 |
| *AK5* | Sanders et al., 2012 |
| *ALDH5A1* | Betancur et al., 2011, Pinto et al., 2010, SFARI database |
| *ALDH7A1* | Betancur et al., 2011 |
| *ALOX12B* | Neale et al., 2012 |
| *ALOX5AP* | SFARI database |
| *AMY2B* | O'Roak et al., 2012 |
| *ANK3* | Sanders et al., 2012, SFARI database |
| *ANKRD11* | Pinto et al., 2010, SFARI database |
| *ANKRD12* | Pinto et al., 2010 |
| *ANKRD35* | Sanders et al., 2012 |
| *ANKS1A* | Neale et al., 2012 |
| *ANTXR1* | Neale et al., 2012 |
| *AOC3* | Neale et al., 2012 |
| *AP1S2* | Betancur et al., 2011 |
| *AP3B2* | O'Roak et al., 2012 |
| *APAF1* | O'Roak et al., 2012 |
| *APBA2* | SFARI database |
| *APC* | SFARI database |
| *APH1A* | Neale et al., 2012 |
| *APLF* | O'Roak et al., 2012 |
| *APLNR* | Neale et al., 2012 |
| *APOC3* | Neale et al., 2012 |
| *AR* | SFARI database |
| *ARHGAP11B* | SFARI database |
| *ARHGAP15* | SFARI database |
| *ARHGAP24* | SFARI database |
| *ARHGEF10L* | O'Roak et al., 2012 |
| *ARHGEF6* | Betancur et al., 2011 |
| *ARID1B* | O'Roak et al., 2012, SFARI database |
| *ARMC9* | O'Roak et al., 2012 |
| *ARNT* | O'Roak et al., 2012 |
| *ARNT2* | Neale et al., 2012, SFARI database |
| *ARX* | Betancur et al., 2011, Pinto et al., 2010, SFARI database |
| *ASAH2* | O'Roak et al., 2012 |
| *ASB1* | Neale et al., 2012 |
| *ASMT* | SFARI database |
| *ASS1* | SFARI database |
| *ASTN1* | Pinto et al., 2010 |
| *ASTN2* | Pinto et al., 2010, SFARI database |
| *ATP10A* | SFARI database |
| *ATP2B2* | SFARI database |
| *ATP2B4* | Sanders et al., 2012 |
| *ATRNL1* | SFARI database |
| *ATRX* | Betancur et al., 2011, Pinto et al., 2010, SFARI database |
| *ATXN7* | SFARI database |
| *AUTS2* | Pinto et al., 2010, SFARI database |
| *AVPR1A* | Pinto et al., 2010, SFARI database |
| *B3GALT6* | Pinto et al., 2010 |
| *BAIAP2* | SFARI database |
| *BCKDK* | SFARI database |
| *BCL2* | SFARI database |
| *BCORL1* | Sanders et al., 2012 |
| *BIN1* | SFARI database |
| *BMP1* | O'Roak et al., 2012 |
| *BRAF* | Betancur et al., 2011 |
| *BRCA2* | Neale et al., 2012, SFARI database |
| *BRD1* | Sanders et al., 2012 |
| *BRSK2* | O'Roak et al., 2012 |
| *BRWD1* | O'Roak et al., 2012 |
| *BSPRY* | O'Roak et al., 2012 |
| *BTAF1* | SFARI database |
| *BTD* | Betancur et al., 2011 |
| *BTN1A1* | Sanders et al., 2012 |
| *BZRAP1* | Pinto et al., 2010, SFARI database |
| *C10orf90* | Neale et al., 2012 |
| *C12orf41* | Neale et al., 2012 |
| *C15orf43* | SFARI database |
| *C15orf62* | O'Roak et al., 2012 |
| *C18orf26* | Sanders et al., 2012 |
| *C1orf125* | Neale et al., 2012 |
| *C20orf111* | Neale et al., 2012 |
| *C2orf42* | Sanders et al., 2012 |
| *C3* | Neale et al., 2012 |
| *C3orf38* | O'Roak et al., 2012 |
| *C3orf58* | Pinto et al., 2010, SFARI database |
| *C4B* | SFARI database |
| *C6orf174* | Sanders et al., 2012 |
| *C7orf43* | Neale et al., 2012 |
| *C9orf144B* | Sanders et al., 2012 |
| *C9orf30* | O'Roak et al., 2012 |
| *C9orf68* | O'Roak et al., 2012 |
| *CA6* | Pinto et al., 2010, SFARI database |
| *CACHD1* | Neale et al., 2012 |
| *CACNA1A* | O'Roak et al., 2012 |
| *CACNA1B* | SFARI database |
| *CACNA1C* | Betancur et al., 2011, Pinto et al., 2010, SFARI database |
| *CACNA1D* | O'Roak et al., 2012, SFARI database |
| *CACNA1E* | Neale et al., 2012, O'Roak et al., 2012 |
| *CACNA1F* | Betancur et al., 2011, Pinto et al., 2010, SFARI database |
| *CACNA1G* | SFARI database |
| *CACNA1H* | SFARI database |
| *CACNA2D2* | O'Roak et al., 2012 |
| *CADM1* | SFARI database |
| *CADPS* | Neale et al., 2012 |
| *CADPS2* | Pinto et al., 2010, SFARI database |
| *CAMSAP2* | SFARI database |
| *CAMTA1* | SFARI database |
| *CAP2* | Pinto et al., 2010 |
| *CAPN10* | Sanders et al., 2012 |
| *CARKD* | O'Roak et al., 2012 |
| *CASC4* | SFARI database |
| *CASK* | Betancur et al., 2011, Pinto et al., 2010, Sanders et al., 2012, SFARI database |
| *CBS* | SFARI database |
| *CCDC14* | Sanders et al., 2012 |
| *CCDC18* | Neale et al., 2012 |
| *CCDC64* | SFARI database |
| *CCDC90B* | Neale et al., 2012 |
| *CCNJ* | Neale et al., 2012 |
| *CD151* | Sanders et al., 2012 |
| *CD247* | Neale et al., 2012 |
| *CD38* | SFARI database |
| *CD44* | SFARI database |
| *CD72* | O'Roak et al., 2012 |
| *CD83* | Pinto et al., 2010 |
| *CD99L2* | SFARI database |
| *CDC42BPB* | O'Roak et al., 2012 |
| *CDCA7L* | Neale et al., 2012 |
| *CDH10* | Pinto et al., 2010, SFARI database |
| *CDH18* | Pinto et al., 2010 |
| *CDH22* | SFARI database |
| *CDH5* | O'Roak et al., 2012 |
| *CDH8* | SFARI database |
| *CDH9* | Pinto et al., 2010, SFARI database |
| *CDHR5* | O'Roak et al., 2012 |
| *CDKL5* | Betancur et al., 2011, Pinto et al., 2010, SFARI database |
| *CDON* | O'Roak et al., 2012 |
| *CDX2* | Neale et al., 2012 |
| *CECR1* | Neale et al., 2012 |
| *CELA1* | O'Roak et al., 2012 |
| *CENTG2* | SFARI database |
| *CEP290 (NPHP6)* | Betancur et al., 2011 |
| *CEP350* | O'Roak et al., 2012 |
| *CEP41* | SFARI database |
| *CHD1* | Neale et al., 2012 |
| *CHD2* | Neale et al., 2012 |
| *CHD3* | O'Roak et al., 2012 |
| *CHD7* | Betancur et al., 2011, Pinto et al., 2010, O'Roak et al., 2012, SFARI database |
| *CHD8* | O'Roak et al., 2012, SFARI database |
| *CHRNA7* | SFARI database |
| *CHRND* | Sanders et al., 2012 |
| *CHST5* | SFARI database |
| *CIAO1* | Neale et al., 2012 |
| *CISH* | O'Roak et al., 2012 |
| *CLTC* | Neale et al., 2012 |
| *CLTCL1* | SFARI database |
| *CMIP* | SFARI database |
| *CNOT1* | O'Roak et al., 2012 |
| *CNOT3* | O'Roak et al., 2012 |
| *CNOT4* | O'Roak et al., 2012 |
| *CNR1* | SFARI database |
| *CNST* | O'Roak et al., 2012 |
| *CNTN3* | Pinto et al., 2010, SFARI database |
| *CNTN4* | Pinto et al., 2010, SFARI database |
| *CNTN5* | SFARI database |
| *CNTN6* | SFARI database |
| *CNTNAP2* | Betancur et al., 2011, Pinto et al., 2010, SFARI database |
| *CNTNAP3* | SFARI database |
| *CNTNAP5* | SFARI database |
| *COL12A1* | O'Roak et al., 2012 |
| *COL15A1* | Neale et al., 2012 |
| *COL25A1* | O'Roak et al., 2012 |
| *CPZ* | Sanders et al., 2012 |
| *CREBBP* | Betancur et al., 2011, Pinto et al., 2010, SFARI database |
| *CSDE1* | Sanders et al., 2012 |
| *CSMD1* | Sanders et al., 2012 |
| *CSNK1D* | SFARI database |
| *CST9* | Neale et al., 2012 |
| *CTNNA3* | SFARI database |
| *CTNNB1* | O'Roak et al., 2012, SFARI database |
| *CTNND2* | Pinto et al., 2010 |
| *CTTNBP2* | SFARI database |
| *CUBN* | O'Roak et al., 2012 |
| *CUL3* | O'Roak et al., 2012, SFARI database |
| *CUL5* | O'Roak et al., 2012 |
| *CXCR3* | SFARI database |
| *CYFIP1* | SFARI database |
| *CYP11B1* | SFARI database |
| *CYP1A2* | Sanders et al., 2012 |
| *CYP20A1* | Neale et al., 2012 |
| *CYP4F3* | Sanders et al., 2012 |
| *CYTH4* | Neale et al., 2012 |
| *DAB1* | SFARI database |
| *DAB2* | Neale et al., 2012 |
| *DAG1* | Neale et al., 2012 |
| *DAPK1* | SFARI database |
| *DBNL* | Sanders et al., 2012 |
| *DBR1* | O'Roak et al., 2012 |
| *DCAF4* | Neale et al., 2012 |
| *DCTN5* | SFARI database |
| *DCUN1D1* | SFARI database |
| *DCX* | Betancur et al., 2011, Pinto et al., 2010, SFARI database |
| *DDC* | SFARI database |
| *DDI2* | O'Roak et al., 2012 |
| *DDR2* | O'Roak et al., 2012 |
| *DDX11* | SFARI database |
| *DDX20* | O'Roak et al., 2012 |
| *DDX53* | SFARI database |
| *DENND5A* | Neale et al., 2012 |
| *DENR* | Neale et al., 2012 |
| *DEPDC7* | O'Roak et al., 2012 |
| *DET1* | O'Roak et al., 2012 |
| *DGCR14* | Sanders et al., 2012 |
| *DGCR8* | O'Roak et al., 2012 |
| *DHCR7* | Betancur et al., 2011, Pinto et al., 2010, SFARI database |
| *DHRS4L1* | O'Roak et al., 2012 |
| *DIAPH3* | SFARI database |
| *DICER1* | Sanders et al., 2012 |
| *DISC1* | Pinto et al., 2010, Sanders et al., 2012, SFARI database |
| *DLG4* | SFARI database |
| *DLGAP2* | Pinto et al., 2010, SFARI database |
| *DLX1* | SFARI database |
| *DLX2* | SFARI database |
| *DLX6* | SFARI database |
| *DMD* | Betancur et al., 2011, Pinto et al., 2010, SFARI database |
| *DMPK* | Betancur et al., 2011, Pinto et al., 2010, SFARI database |
| *DNAH11* | Sanders et al., 2012 |
| *DNAH17* | O'Roak et al., 2012 |
| *DNAH5* | O'Roak et al., 2012, Sanders et al., 2012 |
| *DNAH7* | Neale et al., 2012 |
| *DNAH9* | Sanders et al., 2012 |
| *DNAJB9* | O'Roak et al., 2012 |
| *DNAJC19* | SFARI database |
| *DNER* | SFARI database |
| *DNM1L* | SFARI database |
| *DNMT3A* | Sanders et al., 2012 |
| *DOM3Z* | Sanders et al., 2012 |
| *DPP10* | Pinto et al., 2010, SFARI database |
| *DPP3* | Neale et al., 2012 |
| *DPP4* | O'Roak et al., 2012 |
| *DPP6* | Pinto et al., 2010, SFARI database |
| *DPYD* | Pinto et al., 2010, SFARI database |
| *DRD2* | SFARI database |
| *DRD3* | SFARI database |
| *DUOXA1* | Pinto et al., 2010 |
| *DUS1L* | O'Roak et al., 2012 |
| *DUSP15* | Neale et al., 2012 |
| *DUSP22* | SFARI database |
| *DUSP3* | Neale et al., 2012 |
| *DYRK1A* | O'Roak et al., 2012, SFARI database |
| *EBAG9* | Sanders et al., 2012 |
| *EFR3A* | Sanders et al., 2012 |
| *EGR2* | SFARI database |
| *EHD2* | O'Roak et al., 2012 |
| *EHMT1* | Betancur et al., 2011, Pinto et al., 2010, SFARI database |
| *EIF2C1* | Sanders et al., 2012 |
| *EIF3G* | O'Roak et al., 2012 |
| *EIF4E* | Pinto et al., 2010, SFARI database |
| *EIF4EBP2* | SFARI database |
| *EIF4G1* | O'Roak et al., 2012 |
| *ELK1* | Neale et al., 2012 |
| *ELL* | Neale et al., 2012 |
| *EML1* | SFARI database |
| *EN2* | Pinto et al., 2010, SFARI database |
| *EP300* | SFARI database |
| *EP400* | SFARI database |
| *EPC2* | SFARI database |
| *EPHA6* | SFARI database |
| *EPHB2* | Sanders et al., 2012, SFARI database |
| *EPHB6* | SFARI database |
| *ERBB4* | SFARI database |
| *ERG* | SFARI database |
| *ERV3-1* | O'Roak et al., 2012 |
| *ESR1* | SFARI database |
| *ESR2* | SFARI database |
| *ESRRB* | SFARI database |
| *ETFB* | O'Roak et al., 2012 |
| *EWSR1* | Neale et al., 2012 |
| *EXT1* | SFARI database |
| *F13A1* | SFARI database |
| *FABP5* | SFARI database |
| *FABP7* | SFARI database |
| *FAF2* | O'Roak et al., 2012 |
| *FAM135B* | SFARI database |
| *FAM45A* | O'Roak et al., 2012 |
| *FAM63B* | Neale et al., 2012 |
| *FAM8A1* | Sanders et al., 2012 |
| *FAT1* | Neale et al., 2012, SFARI database |
| *FBLN7* | Neale et al., 2012 |
| *FBXL5* | Neale et al., 2012 |
| *FBXL6* | Sanders et al., 2012 |
| *FBXO10* | O'Roak et al., 2012 |
| *FBXO15* | SFARI database |
| *FBXO18* | Sanders et al., 2012 |
| *FBXO33* | SFARI database |
| *FBXO40* | Pinto et al., 2010, SFARI database |
| *FBXW9* | O'Roak et al., 2012 |
| *FCRL6* | Sanders et al., 2012 |
| *FER* | SFARI database |
| *FEZF2* | Sanders et al., 2012, SFARI database |
| *FGD1* | Betancur et al., 2011, Pinto et al., 2010, SFARI database |
| *FGD3* | Sanders et al., 2012 |
| *FGF22* | Neale et al., 2012 |
| *FGFBP3* | SFARI database |
| *FGFR2* | Betancur et al., 2011 |
| *FHIT* | Pinto et al., 2010, SFARI database |
| *FLT1* | SFARI database |
| *FMR1* | Betancur et al., 2011, Pinto et al., 2010, SFARI database |
| *FN1* | Neale et al., 2012 |
| *FNIP1* | Neale et al., 2012 |
| *FOLH1* | SFARI database |
| *FOXG1* | Betancur et al., 2011, Pinto et al., 2010, SFARI database |
| *FOXP1* | Betancur et al., 2011, Pinto et al., 2010, SFARI database |
| *FOXP2* | SFARI database |
| *FREM3* | Sanders et al., 2012 |
| *FRK* | SFARI database |
| *FRMPD4* | SFARI database |
| *FRYL* | O'Roak et al., 2012 |
| *FTSJ1* | Betancur et al., 2011 |
| *GABRA3* | SFARI database |
| *GABRA4* | Pinto et al., 2010, SFARI database |
| *GABRA5* | Pinto et al., 2010 |
| *GABRB1* | Pinto et al., 2010, SFARI database |
| *GABRB3* | Pinto et al., 2010, SFARI database |
| *GABRG1* | Pinto et al., 2010 |
| *GABRQ* | SFARI database |
| *GAD1* | O'Roak et al., 2012 |
| *GAK* | Neale et al., 2012 |
| *GALC* | Sanders et al., 2012 |
| *GALNT13* | Pinto et al., 2010, SFARI database |
| *GALNT14* | SFARI database |
| *GALNT9* | Pinto et al., 2010 |
| *GAMT* | Betancur et al., 2011 |
| *GAN* | SFARI database |
| *GAP43* | SFARI database |
| *GAS2* | SFARI database |
| *GATA3* | Pinto et al., 2010 |
| *GATM (AGAT)* | Betancur et al., 2011 |
| *GBP6* | Sanders et al., 2012 |
| *GJA10* | O'Roak et al., 2012 |
| *GLDC* | Neale et al., 2012 |
| *GLMN* | O'Roak et al., 2012 |
| *GLO1* | SFARI database |
| *GLRA2* | SFARI database |
| *GMFG* | O'Roak et al., 2012 |
| *GNA11* | O'Roak et al., 2012 |
| *GNA14* | SFARI database |
| *GNAS* | Sanders et al., 2012, SFARI database |
| *GNB1L* | SFARI database |
| *GNL3L* | O'Roak et al., 2012 |
| *GNRHR* | Neale et al., 2012 |
| *GOLGA3* | Sanders et al., 2012 |
| *GON4L* | Sanders et al., 2012 |
| *GPC6* | SFARI database |
| *GPR114* | O'Roak et al., 2012 |
| *GPR139* | Sanders et al., 2012, SFARI database |
| *GPR146* | O'Roak et al., 2012 |
| *GPR89A* | Pinto et al., 2010 |
| *GPR98* | Sanders et al., 2012 |
| *GPRIN3* | Neale et al., 2012 |
| *GPS1* | O'Roak et al., 2012 |
| *GPX1* | SFARI database |
| *GPX8* | Neale et al., 2012 |
| *GREB1L* | Sanders et al., 2012 |
| *GRIA3* | Betancur et al., 2011 |
| *GRID1* | Pinto et al., 2010, SFARI database |
| *GRID2* | SFARI database |
| *GRIK2* | Pinto et al., 2010, SFARI database |
| *GRIK5* | Sanders et al., 2012 |
| *GRIN2A* | Pinto et al., 2010, SFARI database |
| *GRIN2B* | SFARI database |
| *GRIN3B* | O'Roak et al., 2012 |
| *GRIP1* | SFARI database |
| *GRM5* | SFARI database |
| *GRM7* | Sanders et al., 2012 |
| *GRM8* | Pinto et al., 2010, SFARI database |
| *GRPR* | Pinto et al., 2010, SFARI database |
| *GSK3B* | SFARI database |
| *GSN* | SFARI database |
| *GSTM1* | SFARI database |
| *GTF2I* | SFARI database |
| *GTF2IRD1* | Sanders et al., 2012 |
| *GUCY1A2* | SFARI database |
| *GUCY2D* | Betancur et al., 2011 |
| *H2AFV* | O'Roak et al., 2012 |
| *HCFC1* | SFARI database |
| *HDAC4* | SFARI database |
| *HDAC6* | SFARI database |
| *HDGFRP2* | O'Roak et al., 2012 |
| *HDLBP* | O'Roak et al., 2012 |
| *HEPACAM* | SFARI database |
| *HERC2* | SFARI database |
| *HIGD2A* | Sanders et al., 2012 |
| *HIST1H2AE* | Sanders et al., 2012 |
| *HLA-A* | SFARI database |
| *HLA-DRB1* | SFARI database |
| *HLTF* | O'Roak et al., 2012 |
| *HMGN1* | SFARI database |
| *HMHA1* | Sanders et al., 2012 |
| *HNRNPF* | O'Roak et al., 2012 |
| *HNRNPH2* | SFARI database |
| *HOMER1* | SFARI database |
| *HOXA1* | Betancur et al., 2011, Pinto et al., 2010, SFARI database |
| *HOXB1* | SFARI database |
| *HRAS* | Betancur et al., 2011, Pinto et al., 2010, SFARI database |
| *HS3ST5* | SFARI database |
| *HSD11B1* | SFARI database |
| *HSPB2* | O'Roak et al., 2012 |
| *HTATIP2* | O'Roak et al., 2012 |
| *HTR1B* | SFARI database |
| *HTR2A* | SFARI database |
| *HTR3A* | Pinto et al., 2010, SFARI database |
| *HTR3C* | Pinto et al., 2010, SFARI database |
| *HTR7* | SFARI database |
| *HUWE1* | SFARI database |
| *ICA1* | SFARI database |
| *IGF2* | Betancur et al., 2011 |
| *IKBIP* | Sanders et al., 2012 |
| *IL1R2* | Sanders et al., 2012, SFARI database |
| *IL1RAPL1* | Betancur et al., 2011, Pinto et al., 2010, SFARI database |
| *IL1RAPL2* | SFARI database |
| *IL20RB* | Sanders et al., 2012 |
| *IMMP2L* | SFARI database |
| *INCENP* | O'Roak et al., 2012 |
| *INMT* | Neale et al., 2012 |
| *INPP1* | Pinto et al., 2010, SFARI database |
| *INPP5B* | O'Roak et al., 2012 |
| *INTU* | Neale et al., 2012 |
| *IQCE* | Neale et al., 2012 |
| *IQGAP2* | O'Roak et al., 2012 |
| *IQGAP3* | Sanders et al., 2012 |
| *IQSEC2* | Betancur et al., 2011 |
| *IRF2BPL* | O'Roak et al., 2012 |
| *ISLR2* | O'Roak et al., 2012 |
| *ITGA11* | Sanders et al., 2012 |
| *ITGA2* | O'Roak et al., 2012 |
| *ITGA4* | Pinto et al., 2010, SFARI database |
| *ITGA5* | Neale et al., 2012 |
| *ITGB2* | Neale et al., 2012 |
| *ITGB3* | Pinto et al., 2010, O'Roak et al., 2012, SFARI database |
| *ITGB7* | SFARI database |
| *ITSN2* | Sanders et al., 2012 |
| *JARID1C* | Betancur et al., 2011 |
| *JARID2* | SFARI database |
| *JMJD1C* | Pinto et al., 2010, Neale et al., 2012, SFARI database |
| *KALRN* | O'Roak et al., 2012 |
| *KANK1* | Sanders et al., 2012 |
| *KATNAL2* | O'Roak et al., 2012, Sanders et al., 2012SFARI database |
| *KCND2* | SFARI database |
| *KCNJ10* | SFARI database |
| *KCNMA1* | Pinto et al., 2010, Neale et al., 2012, SFARI database |
| *KCNS1* | Neale et al., 2012 |
| *KCNT1* | SFARI database |
| *KCTD13* | SFARI database |
| *KDM5C* | SFARI database |
| *KHDRBS2* | SFARI database |
| *KIAA0100* | O'Roak et al., 2012, Sanders et al., 2012 |
| *KIAA0182* | O'Roak et al., 2012, Sanders et al., 2012 |
| *KIAA0317* | Sanders et al., 2012 |
| *KIAA0319* | O'Roak et al., 2012 |
| *KIAA0556* | Sanders et al., 2012 |
| *KIAA1432* | Sanders et al., 2012 |
| *KIAA1462* | Sanders et al., 2012 |
| *KIAA1549* | Neale et al., 2012 |
| *KIAA1586* | Pinto et al., 2010, SFARI database |
| *KIAA1949* | Neale et al., 2012 |
| *KIAA1967* | Neale et al., 2012 |
| *KIAA2022* | Betancur et al., 2011 |
| *KIF5C* | SFARI database |
| *KIRREL3* | Neale et al., 2012 |
| *KLC2* | SFARI database |
| *KLHL3* | Pinto et al., 2010 |
| *KRAS* | Betancur et al., 2011 |
| *KRBA1* | O'Roak et al., 2012 |
| *KRT80* | O'Roak et al., 2012 |
| *KRTAP9-3* | Neale et al., 2012 |
| *L1CAM* | Betancur et al., 2011 |
| *L1TD1* | O'Roak et al., 2012 |
| *L2HGDH* | Betancur et al., 2011 |
| *L3MBTL1* | Sanders et al., 2012 |
| *LAMA1* | SFARI database |
| *LAMB1* | Pinto et al., 2010, SFARI database |
| *LAMB2* | O'Roak et al., 2012 |
| *LAMC3* | SFARI database |
| *LAMP2* | Betancur et al., 2011 |
| *LASS6* | Sanders et al., 2012 |
| *LCN10* | Sanders et al., 2012 |
| *LGR4* | Neale et al., 2012, O'Roak et al., 2012 |
| *LIPE* | O'Roak et al., 2012 |
| *LLGL1* | Sanders et al., 2012 |
| *LMX1B* | SFARI database |
| *LPHN1* | Sanders et al., 2012 |
| *LRFN5* | SFARI database |
| *LRP1* | O'Roak et al., 2012, Sanders et al., 2012 |
| *LRP2* | SFARI database |
| *LRPPRC* | SFARI database |
| *LRRC1* | SFARI database |
| *LRRC7* | SFARI database |
| *LRRC8B* | O'Roak et al., 2012 |
| *LRRIQ1* | Sanders et al., 2012 |
| *LRRK1* | O'Roak et al., 2012 |
| *LTBP1* | Sanders et al., 2012 |
| *LTN1* | O'Roak et al., 2012 |
| *LY6G6F* | Neale et al., 2012 |
| *LZTS2* | SFARI database |
| *MACC1* | Neale et al., 2012 |
| *MACROD2* | SFARI database |
| *MADD* | Sanders et al., 2012 |
| *MAGED1* | SFARI database |
| *MANSC1* | Neale et al., 2012 |
| *MAOA* | SFARI database |
| *MAP2* | SFARI database |
| *MAP2K1 (MEK1)* | Betancur et al., 2011 |
| *MAP4* | O'Roak et al., 2012 |
| *MAPK1* | SFARI database |
| *MAPK3* | SFARI database |
| *MAPK8IP2* | SFARI database |
| *MARK1* | SFARI database |
| *MARK2* | Sanders et al., 2012 |
| *MBD1* | SFARI database |
| *MBD3* | SFARI database |
| *MBD4* | SFARI database |
| *MBD5* | Betancur et al., 2011, Pinto et al., 2010, O'Roak et al., 2012, SFARI database |
| *MBD6* | SFARI database |
| *MCAM* | O'Roak et al., 2012 |
| *MCC* | SFARI database |
| *MCM2* | Neale et al., 2012 |
| *MCPH1* | Neale et al., 2012, SFARI database |
| *MDGA2* | Pinto et al., 2010, SFARI database |
| *MDH1B* | Neale et al., 2012 |
| *MDM2* | O'Roak et al., 2012 |
| *MDN1* | Sanders et al., 2012 |
| *MECP2* | Betancur et al., 2011, Pinto et al., 2010, SFARI database |
| *MED12* | Betancur et al., 2011, Pinto et al., 2010, SFARI database |
| *MEF2C* | Betancur et al., 2011, Pinto et al., 2010, Neale et al., 2012, SFARI database |
| *MEGF11* | O'Roak et al., 2012, Sanders et al., 2012 |
| *MET* | Pinto et al., 2010, SFARI database |
| *METTL14* | Neale et al., 2012 |
| *METTL20* | O'Roak et al., 2012 |
| *MICALCL* | Neale et al., 2012 |
| *MID1* | Betancur et al., 2011 |
| *MKI67* | O'Roak et al., 2012 |
| *MKKS* | Betancur et al., 2011 |
| *MKL2* | Neale et al., 2012, SFARI database |
| *MLL* | Neale et al., 2012 |
| *MLL3* | O'Roak et al., 2012 |
| *MMP8* | Sanders et al., 2012 |
| *MMRN2* | Neale et al., 2012 |
| *MOBKL3* | Neale et al., 2012 |
| *MPDZ* | Neale et al., 2012 |
| *MPHOSPH8* | Sanders et al., 2012 |
| *MPP6* | Sanders et al., 2012 |
| *MR1* | Sanders et al., 2012 |
| *MSH6* | O'Roak et al., 2012 |
| *MSN* | SFARI database |
| *MSR1* | SFARI database |
| *MST1R* | O'Roak et al., 2012 |
| *MTF1* | Pinto et al., 2010, SFARI database |
| *MTHFR* | SFARI database |
| *MTMR12* | Neale et al., 2012 |
| *MTMR2* | Sanders et al., 2012 |
| *MTMR9* | Sanders et al., 2012 |
| *MTX2* | SFARI database |
| *MUC16* | O'Roak et al., 2012 |
| *MUC4* | Neale et al., 2012 |
| *MYB* | Sanders et al., 2012 |
| *MYBBP1A* | O'Roak et al., 2012 |
| *MYCBP2* | Neale et al., 2012 |
| *MYH10* | O'Roak et al., 2012 |
| *MYH9* | Sanders et al., 2012 |
| *MYO16* | SFARI database |
| *MYO1A* | SFARI database |
| *MYO5B* | Neale et al., 2012 |
| *MYO7A* | Neale et al., 2012 |
| *MYO7B* | O'Roak et al., 2012, Sanders et al., 2012 |
| *MYOF* | Neale et al., 2012 |
| *MYOM2* | Neale et al., 2012 |
| *NAA40* | O'Roak et al., 2012 |
| *NAB2* | Neale et al., 2012 |
| *NACA* | O'Roak et al., 2012 |
| *NAPRT1* | Sanders et al., 2012 |
| *NAV2* | O'Roak et al., 2012, Sanders et al., 2012 |
| *NBEA* | Pinto et al., 2010, SFARI database |
| *NBEAL1* | Sanders et al., 2012 |
| *NCAPD2* | Sanders et al., 2012 |
| *NCKAP5L* | SFARI database |
| *NDNL2* | Pinto et al., 2010, SFARI database |
| *NDP* | Betancur et al., 2011 |
| *NDST4* | O'Roak et al., 2012 |
| *NDUFA5* | SFARI database |
| *NDUFV3* | Pinto et al., 2010 |
| *NEFL* | SFARI database |
| *NEGR1* | Pinto et al., 2010 |
| *NELL1* | SFARI database |
| *NF1* | Betancur et al., 2011, Pinto et al., 2010, Sanders et al., 2012, SFARI database |
| *NFASC* | Neale et al., 2012 |
| *NFIA* | SFARI database |
| *NFIX* | Betancur et al., 2011 |
| *NHS* | Betancur et al., 2011 |
| *NIPA1* | SFARI database |
| *NIPA2* | SFARI database |
| *NIPBL* | Betancur et al., 2011, Pinto et al., 2010, SFARI database |
| *NISCH* | Neale et al., 2012 |
| *NLGN1* | Pinto et al., 2010, O'Roak et al., 2012, SFARI database |
| *NLGN3* | Betancur et al., 2011, Pinto et al., 2010, SFARI database |
| *NLGN4X* | Betancur et al., 2011, Pinto et al., 2010, SFARI database |
| *NLGN4Y* | SFARI database |
| *NLRP11* | Sanders et al., 2012 |
| *NLRX1* | Sanders et al., 2012 |
| *NOLC1* | O'Roak et al., 2012 |
| *NOS1AP* | SFARI database |
| *NOS2A* | SFARI database |
| *NOTCH3* | O'Roak et al., 2012 |
| *NPAS2* | SFARI database |
| *NPFFR2* | Sanders et al., 2012 |
| *NPHP1* | Betancur et al., 2011 |
| *NR2F1* | Sanders et al., 2012 |
| *NR4A2* | O'Roak et al., 2012 |
| *NR5A2* | Neale et al., 2012 |
| *NRCAM* | Pinto et al., 2010, SFARI database |
| *NRP2* | SFARI database |
| *NRXN1* | Betancur et al., 2011, Pinto et al., 2010, SFARI database |
| *NRXN2* | SFARI database |
| *NRXN3* | SFARI database |
| *NSD1* | Betancur et al., 2011, Pinto et al., 2010, SFARI database |
| *NSUN7* | Sanders et al., 2012 |
| *NTNG1* | O'Roak et al., 2012, SFARI database |
| *NTRK1* | SFARI database |
| *NTRK3* | SFARI database |
| *NUP133* | O'Roak et al., 2012 |
| *NUP98* | Neale et al., 2012 |
| *NXF5* | SFARI database |
| *NXPH1* | SFARI database |
| *OBSL1* | Neale et al., 2012 |
| *OCRL* | Betancur et al., 2011 |
| *ODF3L2* | SFARI database |
| *OPHN1* | Betancur et al., 2011, Pinto et al., 2010, SFARI database |
| *OPLAH* | O'Roak et al., 2012 |
| *OPRL1* | O'Roak et al., 2012 |
| *OPRM1* | SFARI database |
| *OR10Z1* | O'Roak et al., 2012 |
| *OR11L1* | O'Roak et al., 2012 |
| *OR1C1* | SFARI database |
| *OR2D2* | O'Roak et al., 2012 |
| *OR5AC2* | Sanders et al., 2012 |
| *OR6N2* | Sanders et al., 2012 |
| *OR8H1* | Neale et al., 2012 |
| *OTC* | Betancur et al., 2011 |
| *OTX1* | SFARI database |
| *OXTR* | Pinto et al., 2010, SFARI database |
| *PACS2* | O'Roak et al., 2012 |
| *PAEP* | O'Roak et al., 2012 |
| *PAFAH1B1* | Betancur et al., 2011 |
| *PAH* | Betancur et al., 2011 |
| *PAK7* | Pinto et al., 2010 |
| *PAPPA2* | Pinto et al., 2010 |
| *PAQR4* | O'Roak et al., 2012 |
| *ParAngton* | Pinto et al., 2010 |
| *PARD3B* | SFARI database |
| *PARK2* | Pinto et al., 2010, SFARI database |
| *PBRM1* | O'Roak et al., 2012 |
| *PCDH10* | Pinto et al., 2010, SFARI database |
| *PCDH15* | SFARI database |
| *PCDH19* | Betancur et al., 2011, Pinto et al., 2010, SFARI database |
| *PCDH8* | SFARI database |
| *PCDH9* | Pinto et al., 2010, SFARI database |
| *PCDHA1* | SFARI database |
| *PCDHA10* | SFARI database |
| *PCDHA11* | SFARI database |
| *PCDHA12* | SFARI database |
| *PCDHA13* | SFARI database |
| *PCDHA2* | SFARI database |
| *PCDHA3* | SFARI database |
| *PCDHA4* | SFARI database |
| *PCDHA5* | SFARI database |
| *PCDHA6* | SFARI database |
| *PCDHA7* | SFARI database |
| *PCDHA8* | SFARI database |
| *PCDHA9* | SFARI database |
| *PCDHAC1* | SFARI database |
| *PCDHAC2* | SFARI database |
| *PCDHB16* | Neale et al., 2012 |
| *PCDHB4* | O'Roak et al., 2012 |
| *PCDHGA11* | SFARI database |
| *PCNX* | O'Roak et al., 2012 |
| *PDCD1* | O'Roak et al., 2012 |
| *PDE1C* | SFARI database |
| *PDE4A* | SFARI database |
| *PDE4B* | SFARI database |
| *PDE9A* | Pinto et al., 2010 |
| *PDIA6* | O'Roak et al., 2012 |
| *PDZD4* | SFARI database |
| *PECR* | SFARI database |
| *PER1* | Neale et al., 2012, SFARI database |
| *PEX7* | SFARI database |
| *PGD* | O'Roak et al., 2012 |
| *PHF19* | O'Roak et al., 2012 |
| *PHF3* | Sanders et al., 2012 |
| *PHF6* | Betancur et al., 2011 |
| *PHF8* | Betancur et al., 2011, Pinto et al., 2010, SFARI database |
| *PIAS1* | Neale et al., 2012 |
| *PIK3CG* | Pinto et al., 2010, SFARI database |
| *PINX1* | SFARI database |
| *PION* | O'Roak et al., 2012 |
| *PIP5K1B* | Pinto et al., 2010 |
| *PITPNM3* | O'Roak et al., 2012 |
| *PITX1* | SFARI database |
| *PIWIL4* | Neale et al., 2012 |
| *PKD1L3* | O'Roak et al., 2012 |
| *PKNOX1* | Pinto et al., 2010 |
| *PLAUR* | SFARI database |
| *PLCB1* | SFARI database |
| *PLCD1* | SFARI database |
| *PLCD4* | Neale et al., 2012 |
| *PLEC* | Neale et al., 2012 |
| *PLEKHA8* | O'Roak et al., 2012 |
| *PLN* | Pinto et al., 2010, SFARI database |
| *PLOD3* | Sanders et al., 2012 |
| *PLXDC1* | O'Roak et al., 2012 |
| *PLXNB1* | Neale et al., 2012 |
| *PNPLA7* | O'Roak et al., 2012 |
| *POGZ* | Neale et al., 2012, SFARI database |
| *POLQ* | O'Roak et al., 2012 |
| *POLR2A* | Neale et al., 2012 |
| *POLR2M* | O'Roak et al., 2012 |
| *POLRMT* | O'Roak et al., 2012 |
| *POMGNT1* | Betancur et al., 2011 |
| *POMT1* | Betancur et al., 2011 |
| *PON1* | SFARI database |
| *POPDC2* | Sanders et al., 2012 |
| *PPM1D* | Sanders et al., 2012 |
| *PPP1R15B* | Neale et al., 2012 |
| *PPP1R1B* | SFARI database |
| *PPP1R3F* | SFARI database |
| *PPP2R1B* | Sanders et al., 2012 |
| *PQBP1* | Betancur et al., 2011 |
| *PRCP* | Sanders et al., 2012 |
| *PRKAG2* | Pinto et al., 2010 |
| *PRKCA* | O'Roak et al., 2012 |
| *PRKCB* | Pinto et al., 2010, SFARI database |
| *PRKD1* | SFARI database |
| *PRPF39* | Neale et al., 2012 |
| *PRSS12* | Betancur et al., 2011 |
| *PRSS38* | SFARI database |
| *PRUNE2* | SFARI database |
| *PSD3* | SFARI database |
| *PSEN1* | O'Roak et al., 2012 |
| *PSMD10* | SFARI database |
| *PSMG4* | O'Roak et al., 2012 |
| *PTCHD1* | Betancur et al., 2011, Pinto et al., 2010, SFARI database |
| *PTEN* | Betancur et al., 2011, Pinto et al., 2010, O'Roak et al., 2012, SFARI database |
| *PTGES* | O'Roak et al., 2012 |
| *PTGR1* | O'Roak et al., 2012 |
| *PTGS2* | SFARI database |
| *PTK7* | Sanders et al., 2012 |
| *PTPN11* | Betancur et al., 2011, Pinto et al., 2010, SFARI database |
| *PTPRC* | SFARI database |
| *PTPRK* | O'Roak et al., 2012 |
| *PTPRT* | SFARI database |
| *PWWP2A* | Neale et al., 2012 |
| *RAB11FIP5* | SFARI database |
| *RAB2A* | Sanders et al., 2012 |
| *RAB39B* | Betancur et al., 2011, Pinto et al., 2010, SFARI database |
| *RAI1* | Betancur et al., 2011, Pinto et al., 2010, SFARI database |
| *RAPGEF4* | SFARI database |
| *RASSF5* | SFARI database |
| *RB1CC1* | Pinto et al., 2010, SFARI database |
| *RBFOX1* | SFARI database |
| *RBMS3* | SFARI database |
| *REEP3* | Pinto et al., 2010, SFARI database |
| *RELN* | Neale et al., 2012, SFARI database |
| *REST* | Neale et al., 2012 |
| *RFWD2* | Pinto et al., 2010, SFARI database |
| *RFX2* | Sanders et al., 2012 |
| *RFX8* | O'Roak et al., 2012, Sanders et al., 2012 |
| *RGMA* | O'Roak et al., 2012 |
| *RGS22* | O'Roak et al., 2012 |
| *RGS7* | SFARI database |
| *RHOXF1* | SFARI database |
| *RIMS2* | O'Roak et al., 2012 |
| *RIMS3* | Pinto et al., 2010, SFARI database |
| *RNF123* | O'Roak et al., 2012 |
| *RNF135* | Betancur et al., 2011 |
| *RNF182* | Pinto et al., 2010 |
| *RNF38* | Sanders et al., 2012 |
| *RNF8* | Pinto et al., 2010 |
| *ROBO1* | SFARI database |
| *ROCK1* | O'Roak et al., 2012 |
| *RORA* | SFARI database |
| *ROS1* | Sanders et al., 2012 |
| *RPE65* | Betancur et al., 2011 |
| *RPGRIP1L* | Betancur et al., 2011 |
| *RPL10* | Pinto et al., 2010, SFARI database |
| *RPP25* | SFARI database |
| *RPRD1A* | Sanders et al., 2012 |
| *RPS6KA2* | SFARI database |
| *RPS6KA3* | O'Roak et al., 2012 |
| *RTF1* | Neale et al., 2012 |
| *RTN4RL1* | Neale et al., 2012 |
| *RUVBL1* | O'Roak et al., 2012 |
| *RYR1* | O'Roak et al., 2012 |
| *SAFB* | O'Roak et al., 2012 |
| *SATB2* | Betancur et al., 2011, Pinto et al., 2010, SFARI database |
| *SBF1* | Neale et al., 2012, O'Roak et al., 2012, SFARI database |
| *SCARB2* | Sanders et al., 2012 |
| *SCFD2* | SFARI database |
| *SCHIP1* | O'Roak et al., 2012 |
| *SCN1A* | Betancur et al., 2011, Pinto et al., 2010, SFARI database |
| *SCN2A* | Sanders et al., 2012, SFARI database |
| *SCN7A* | Pinto et al., 2010 |
| *SCN8A* | SFARI database |
| *SCP2* | Sanders et al., 2012 |
| *SCRIB* | Neale et al., 2012 |
| *SCYL1* | O'Roak et al., 2012 |
| *SDC2* | SFARI database |
| *SDK1* | SFARI database |
| *SEC22C* | Sanders et al., 2012 |
| *SEL1L* | Neale et al., 2012 |
| *SEMA4G* | Sanders et al., 2012 |
| *SEMA5A* | Pinto et al., 2010, SFARI database |
| *SERPINE1* | SFARI database |
| *SESN2* | O'Roak et al., 2012 |
| *SETBP1* | O'Roak et al., 2012 |
| *SETD2* | O'Roak et al., 2012, SFARI database |
| *SETD5* | Neale et al., 2012 |
| *SETDB1* | SFARI database |
| *SETDB2* | SFARI database |
| *SEZ6L2* | SFARI database |
| *SFPQ* | O'Roak et al., 2012 |
| *SGSH* | Betancur et al., 2011 |
| *SGSM3* | O'Roak et al., 2012, SFARI database |
| *SH2D3C* | O'Roak et al., 2012 |
| *SH3KBP1* | SFARI database |
| *SHANK1* | Pinto et al., 2010, SFARI database |
| *SHANK2* | Betancur et al., 2011, Pinto et al., 2010, Sanders et al., 2012, SFARI database |
| *SHANK3* | Betancur et al., 2011, Pinto et al., 2010, SFARI database |
| *SLC16A3* | SFARI database |
| *SLC16A7* | SFARI database |
| *SLC17A3* | Sanders et al., 2012 |
| *SLC17A6* | O'Roak et al., 2012 |
| *SLC1A1* | SFARI database |
| *SLC1A2* | Neale et al., 2012 |
| *SLC22A15* | SFARI database |
| *SLC22A9* | O'Roak et al., 2012, Sanders et al., 2012 |
| *SLC25A12* | Pinto et al., 2010, SFARI database |
| *SLC25A14* | SFARI database |
| *SLC25A24* | SFARI database |
| *SLC25A27* | SFARI database |
| *SLC26A5* | Sanders et al., 2012 |
| *SLC30A5* | Sanders et al., 2012, SFARI database |
| *SLC34A3* | O'Roak et al., 2012 |
| *SLC38A10* | SFARI database |
| *SLC39A11* | SFARI database |
| *SLC39A5* | Neale et al., 2012 |
| *SLC40A1* | O'Roak et al., 2012 |
| *SLC4A10* | Pinto et al., 2010, SFARI database |
| *SLC6A1* | Sanders et al., 2012 |
| *SLC6A13* | Sanders et al., 2012 |
| *SLC6A3* | Neale et al., 2012 |
| *SLC6A4* | Pinto et al., 2010, Neale et al., 2012, SFARI database |
| *SLC6A8* | Betancur et al., 2011, Pinto et al., 2010, SFARI database |
| *SLC7A7* | O'Roak et al., 2012 |
| *SLC8A3* | Sanders et al., 2012 |
| *SLC9A1* | O'Roak et al., 2012 |
| *SLC9A6* | Betancur et al., 2011, Pinto et al., 2010, SFARI database |
| *SLC9A9* | Pinto et al., 2010, SFARI database |
| *SLCO1C1* | O'Roak et al., 2012, Sanders et al., 2012 |
| *SLFN5* | Sanders et al., 2012 |
| *SMARCC1* | Neale et al., 2012 |
| *SMARCC2* | Neale et al., 2012 |
| *SMC1A* | Betancur et al., 2011 |
| *SMC3* | Sanders et al., 2012 |
| *SMCHD1* | Neale et al., 2012 |
| *SNAPC5* | Sanders et al., 2012 |
| *SND1* | SFARI database |
| *SNRK* | Sanders et al., 2012 |
| *SNRPN* | SFARI database |
| *SNTG1* | Neale et al., 2012 |
| *SNTG2* | SFARI database |
| *SNX19* | SFARI database |
| *SORCS1* | Sanders et al., 2012 |
| *SOX5* | SFARI database |
| *SP7* | O'Roak et al., 2012 |
| *SPAG17* | Sanders et al., 2012 |
| *SPAST* | Neale et al., 2012, SFARI database |
| *SPP2* | Neale et al., 2012 |
| *SPRR2D* | Neale et al., 2012 |
| *SRBD1* | O'Roak et al., 2012 |
| *SRGAP1* | Neale et al., 2012 |
| *SRGAP3* | Sanders et al., 2012 |
| *SRPK2* | Pinto et al., 2010 |
| *SRPR* | O'Roak et al., 2012 |
| *SRRM2* | O'Roak et al., 2012 |
| *ST3GAL3* | O'Roak et al., 2012 |
| *ST3GAL6* | Neale et al., 2012 |
| *ST7* | Pinto et al., 2010, SFARI database |
| *ST8SIA2* | SFARI database |
| *STARD9* | O'Roak et al., 2012 |
| *STAT2* | Neale et al., 2012 |
| *STIL* | O'Roak et al., 2012 |
| *STK3* | Pinto et al., 2010 |
| *STK36* | O'Roak et al., 2012 |
| *STK39* | SFARI database |
| *STXBP1* | Neale et al., 2012, SFARI database |
| *STXBP3* | Neale et al., 2012 |
| *SUCLA2* | Neale et al., 2012 |
| *SUCLG2* | Pinto et al., 2010, SFARI database |
| *SUPT16H* | Sanders et al., 2012 |
| *SUV420H1* | Sanders et al., 2012, SFARI database |
| *SV2B* | Sanders et al., 2012 |
| *SVIL* | Neale et al., 2012 |
| *SYN1* | Betancur et al., 2011, Pinto et al., 2010, SFARI database |
| *SYNE1* | SFARI database |
| *SYNE2* | Neale et al., 2012 |
| *SYNGAP1* | Betancur et al., 2011, Pinto et al., 2010, SFARI database |
| *SYNRG* | O'Roak et al., 2012 |
| *SYT17* | SFARI database |
| *SYT3* | SFARI database |
| *TAF1C* | SFARI database |
| *TAF1L* | SFARI database |
| *TAS2R10* | O'Roak et al., 2012 |
| *TAS2R3* | Sanders et al., 2012 |
| *TATDN2* | O'Roak et al., 2012 |
| *TBC1D5* | SFARI database |
| *TBL1X* | SFARI database |
| *TBL1XR1* | O'Roak et al., 2012, SFARI database |
| *TBR1* | Neale et al., 2012, O'Roak et al., 2012, SFARI database |
| *TBX1* | Betancur et al., 2011, Pinto et al., 2010, SFARI database |
| *TBX18* | Neale et al., 2012 |
| *TBX4* | Neale et al., 2012 |
| *TCERG1L* | Sanders et al., 2012 |
| *TCF3* | Neale et al., 2012 |
| *TCF4* | O'Roak et al., 2012 |
| *TCF7L1* | Sanders et al., 2012 |
| *TDO2* | Pinto et al., 2010, SFARI database |
| *TDRD5* | Neale et al., 2012 |
| *TECTA* | Sanders et al., 2012 |
| *TEPP* | O'Roak et al., 2012 |
| *TET1* | Sanders et al., 2012 |
| *TGM3* | SFARI database |
| *TH* | SFARI database |
| *THRA* | SFARI database |
| *TIPIN* | O'Roak et al., 2012 |
| *TJP3* | Sanders et al., 2012 |
| *TLK2* | SFARI database |
| *TMC4* | O'Roak et al., 2012 |
| *TMEM195* | Pinto et al., 2010 |
| *TMEM231* | SFARI database |
| *TMEM56* | Neale et al., 2012 |
| *TMEM85* | Sanders et al., 2012 |
| *TMEM8A* | Sanders et al., 2012 |
| *TMLHE* | SFARI database |
| *TMPRSS2* | O'Roak et al., 2012 |
| *TNIP2* | SFARI database |
| *TNKS* | O'Roak et al., 2012 |
| *TOMM20* | SFARI database |
| *TOP1* | Neale et al., 2012 |
| *TOPORS* | Neale et al., 2012 |
| *TPH2* | Pinto et al., 2010, SFARI database |
| *TPK1* | Sanders et al., 2012 |
| *TPO* | SFARI database |
| *TPPP* | Pinto et al., 2010 |
| *TRAF7* | Neale et al., 2012 |
| *TRAPPC8* | O'Roak et al., 2012 |
| *TREH* | O'Roak et al., 2012 |
| *TRIO* | O'Roak et al., 2012, Sanders et al., 2012 |
| *TRIP12* | SFARI database |
| *TROAP* | Sanders et al., 2012 |
| *TRPM5* | O'Roak et al., 2012 |
| *TRPM7* | Sanders et al., 2012 |
| *TRRAP* | Sanders et al., 2012 |
| *TSC1* | Betancur et al., 2011, Pinto et al., 2010, SFARI database |
| *TSC2* | Betancur et al., 2011, Pinto et al., 2010, O'Roak et al., 2012, SFARI database |
| *TSN* | SFARI database |
| *TSNARE1* | O'Roak et al., 2012 |
| *TSPAN17* | O'Roak et al., 2012 |
| *TSPAN7* | SFARI database |
| *TSPYL5* | Sanders et al., 2012 |
| *TSR2* | O'Roak et al., 2012 |
| *TTC28* | O'Roak et al., 2012 |
| *TTC39A* | O'Roak et al., 2012 |
| *TTF2* | Neale et al., 2012 |
| *TTN* | O'Roak et al., 2012, SFARI database |
| *TUBA1A* | Neale et al., 2012, Sanders et al., 2012 |
| *TUBG1* | Sanders et al., 2012 |
| *TUBGCP5* | Sanders et al., 2012, SFARI database |
| *TYR* | SFARI database |
| *UBE2H* | Pinto et al., 2010, SFARI database |
| *UBE3A* | Betancur et al., 2011, Pinto et al., 2010, SFARI database |
| *UBE3B* | SFARI database |
| *UBE3C* | O'Roak et al., 2012, SFARI database |
| *UBL7* | SFARI database |
| *UBR3* | O'Roak et al., 2012 |
| *UBR5* | SFARI database |
| *UBR7* | SFARI database |
| *UGGT1* | O'Roak et al., 2012 |
| *UGT2B10* | Neale et al., 2012 |
| *UIMC1* | O'Roak et al., 2012 |
| *UNK* | Sanders et al., 2012 |
| *UPF1* | Neale et al., 2012 |
| *UPF3B* | Betancur et al., 2011, Pinto et al., 2010, SFARI database |
| *USH2A* | Neale et al., 2012 |
| *USP15* | O'Roak et al., 2012 |
| *USP18* | Sanders et al., 2012 |
| *USP46* | Sanders et al., 2012 |
| *USP9Y* | SFARI database |
| *VASH1* | SFARI database |
| *VAV3* | Neale et al., 2012 |
| *VKORC1L1* | O'Roak et al., 2012 |
| *VPS13B* | Betancur et al., 2011, Pinto et al., 2010, SFARI database |
| *VPS39* | O'Roak et al., 2012 |
| *VWCE* | O'Roak et al., 2012 |
| *WDR4* | Pinto et al., 2010, Sanders et al., 2012 |
| *WDR66* | Neale et al., 2012 |
| *WNK3* | SFARI database |
| *WNT2* | Pinto et al., 2010, SFARI database |
| *WNT5A* | O'Roak et al., 2012 |
| *XIRP1* | SFARI database |
| *XPC* | SFARI database |
| *XPO1* | SFARI database |
| *XPO5* | Neale et al., 2012 |
| *YEATS2* | SFARI database |
| *YME1L1* | Sanders et al., 2012 |
| *YTHDC2* | O'Roak et al., 2012, SFARI database |
| *YWHAE* | Betancur et al., 2011 |
| *ZBED2* | O'Roak et al., 2012 |
| *ZBED4* | O'Roak et al., 2012 |
| *ZBTB16* | SFARI database |
| *ZBTB41* | O'Roak et al., 2012 |
| *ZC3H12B* | O'Roak et al., 2012 |
| *ZDHHC1* | Neale et al., 2012 |
| *ZFC3H1* | Neale et al., 2012 |
| *ZFYVE9* | O'Roak et al., 2012 |
| *ZKSCAN5* | Neale et al., 2012 |
| *ZMYM2* | Neale et al., 2012 |
| *ZMYND8* | O'Roak et al., 2012 |
| *ZNF107* | Neale et al., 2012 |
| *ZNF155* | Neale et al., 2012 |
| *ZNF18* | SFARI database |
| *ZNF213* | Sanders et al., 2012 |
| *ZNF292* | Neale et al., 2012 |
| *ZNF311* | Neale et al., 2012 |
| *ZNF335* | Sanders et al., 2012 |
| *ZNF407* | SFARI database |
| *ZNF420* | O'Roak et al., 2012 |
| *ZNF451* | Neale et al., 2012 |
| *ZNF638* | Neale et al., 2012 |
| *ZNF642* | Sanders et al., 2012 |
| *ZNF644* | O'Roak et al., 2012 |
| *ZNF674* | Betancur et al., 2011 |
| *ZNF676* | Pinto et al., 2010 |
| *ZNF81* | Betancur et al., 2011 |
| *ZNF813* | Neale et al., 2012 |
| *ZNF827* | SFARI database |
| *ZSWIM5* | SFARI database |

| **Supporting Table 4 - Variants Identified in Exome Sequencing and Validated by a Second Platform** | | | | | | | | |
| --- | --- | --- | --- | --- | --- | --- | --- | --- |
|  |  |  |  |  |  |  |  |  |
| **Gene** | **Family** | **Position (Hg19)** | **dbSNP** | **Nucleotide** | **Mutation** | **HIHG control chromosomes** | **HumanExome BeadChip** | **Sanger Sequencing** |
| *A4GNT* | 18074 | chr3:137849888 | rs79791762 | C>T | missense | 21/616 | validated | - |
| *ABCA13* | 7936 | chr7:48273776 | - | C>T | missense | 0/610 | - | validated |
| *ABCA4* | 37425 | chr1:94512565 | rs1801581 | C>T | missense | 19/616 | validated | - |
| *ABCA9* | 37994 | chr17:67023524 | rs79212004 | C>T | missense | 2/614 | validated | - |
| *ABCC1* | 37024 | chr16:16142079 | rs60782127 | G>T | missense | 3/614 | validated | - |
| *ABCD2* | 17342 | chr12:40013392 | rs117275340 | G>C | missense | 10/614 | validated | - |
| *ABHD14A* | 17351 | chr3:52011912 | rs17849626 | G>A | missense | 25/614 | validated | validated |
| *ABHD14A* | 17351 | chr3:52014897 | rs61729088 | C>G | missense | 2/616 | validated | validated |
| *ABI3BP* | 18074 | chr3:100585792 | rs113364496 | C>T | missense | 9/586 | validated | - |
| *ACAP1* | 17545 | chr17:7251713 | rs35019942 | C>T | missense | 7/606 | validated | - |
| *ACN9* | 7797 | chr7:96747192 | rs62624461 | T>C | missense | 22/614 | validated | - |
| *ACOT11* | 17342 | chr1:55050353 | rs41294808 | G>A | missense | 21/614 | validated | - |
| *ACOT12* | 17678 | chr5:80641789 | rs34607174 | C>T | missense | 16/614 | validated | - |
| *ACOX1* | 17678 | chr17:73944496 | rs35629489 | G>A | missense | 1/614 | validated | - |
| *ACSM3/ERI2* | 17678 | chr16:20796338 | rs147257547 | G>A | missense | 1/614 | validated | - |
| *ACSM5* | 37534 | chr16:20442594 | rs138834094 | C>T | missense | 4/614 | validated | - |
| *ACTN3* | 37037 | chr11:66330533 | rs71457732 | C>T | missense | 4/584 | validated | - |
| *ADAMTS13* | 37425 | chr9:136319589 | rs28503257 | G>A | missense | 21/610 | validated | - |
| *ADCY1* | 7936 | chr7:45747949 | rs45444695 | G>A | missense | 12/614 | validated | - |
| *ADRA1A* | 37037 | chr8:26721888 | rs2229125 | A>C | missense | 13/610 | validated | - |
| *ADRA1A* | 7531 | chr8:26722027 | rs61757009 | A>C | missense | 2/612 | validated | - |
| *AGAP1* | 17478 | chr2:236659033 | rs143378661 | G>A | missense | 0/616 | validated | validated |
| *AGBL2* | 37425 | chr11:47711820 | rs76215382 | A>G | missense | 12/614 | validated | - |
| *AGTR1* | 37037 | chr3:148459552 | rs12721225 | G>T | missense | 4/616 | validated | - |
| *AKAP13* | 17122 | chr15:86123851 | rs35624420 | C>T | missense | 20/614 | validated | - |
| *ALOXE3* | 7658 | chr17:8021612 | rs79377087 | C>A | missense | 6/608 | validated | - |
| *ALPPL2* | 7870 | chr2:233272091 | rs146482704 | C>T | missense | 7/614 | validated | - |
| *ALS2CL* | 17545 | chr3:46712490 | rs77367607 | G>A | missense | 20/614 | validated | - |
| *ALS2CL* | 17122 | chr3:46719860 | rs140347863 | T>C | missense | 0/612 | validated | validated |
| *AMDHD2* | 37024 | chr16:2578297 | rs146802053 | C>T | missense | 1/566 | validated | - |
| *ANK2* | 17545 | chr4:114279674 | rs34270799 | C>A | missense | 18/614 | validated | - |
| *ANKRD30A* | 7435 | chr10:37419292 | rs116939015 | G>T | nonsense | 12/612 | - | validated |
| *ANKRD30A* | 37232 | chr10:37508345 | - | G>T | missense | 0/612 | - | validated |
| *ANKS1B* | 17678 | chr12:99837570 | rs148678454 | C>T | missense | 10/602 | validated | - |
| *AP4M1* | 17122 | chr7:99702946 | - | C>T | missense | 0/614 | - | validated |
| *APEX1* | 17245 | chr14:20924167 | rs1048945 | G>C | missense | 19/614 | validated | validated |
| *ARMC4* | 17342 | chr10:28149640 | rs150655393 | C>T | missense | 0/614 | validated | validated |
| *ARRDC4* | 37425 | chr15:98509167 | rs146146673 | G>C | missense | 6/614 | validated | - |
| *ASXL3* | 37425 | chr18:31326012 | rs144534810 | T>G | missense | 5/582 | validated | - |
| *ATP10B* | 7590 | chr5:160042903 | rs61734665 | G>T | missense | 14/612 | validated | - |
| *ATP13A4* | 37994 | chr3:193185128 | rs138509256 | A>G | missense | 7/616 | validated | - |
| *ATP6AP1L* | 37425 | chr5:81608563 | rs61740965 | T>C | missense | 9/614 | validated | - |
| *ATP6V0A2* | 7637 | chr12:124241506 | rs17883456 | C>T | missense | 30/614 | validated | - |
| *ATR* | 17545 | chr3:142281298 | rs28897764 | C>T | missense | 8/616 | validated | - |
| *AVIL* | 7435 | chr12:58201505 | rs150785031 | C>G | missense | 2/614 | validated | validated |
| *B4GALT3* | 37232 | chr1:161141673 | rs41270033 | C>T | missense | 3/616 | validated | - |
| *BCL2L14* | 17351 | chr12:12247836 | rs73053321 | C>T | missense | 10/614 | validated | - |
| *BLM* | 7590 | chr15:91290665 | rs148545569 | C>T | missense | 0/614 | validated | - |
| *BPHL* | 7531 | chr6:3140676 | - | G>A | missense | 0/614 | validated | - |
| *BPHL* | 7531 | chr6:3152781 | rs140507260 | A>G | missense | 0/614 | validated | - |
| *BTN2A2* | 37425 | chr6:26384060 | rs73736234 | C>T | missense | 3/614 | validated | - |
| *BTN2A2* | 37425 | chr6:26385263 | rs57038103 | C>G | missense | 2/614 | validated | - |
| *C10orf12* | 37425 | chr10:98741973 | rs72819869 | A>G | missense | 2/614 | validated | - |
| *C10orf128* | 7936 | chr10:50376004 | rs61748312 | A>G | missense | 28/614 | validated | - |
| *C12orf49* | 7590 | chr12:117175608 | rs73220422 | C>T | missense | 15/612 | validated | - |
| *C12orf65* | 7531 | chr12:123738265 | rs78651634 | G>A | missense | 14/614 | validated | - |
| *C12orf73* | 37994 | chr12:104350408 | rs116995648 | C>T | splicing | 0/592 | validated | - |
| *C14orf102* | 37024 | chr14:90756862 | rs45462994 | C>G | missense | 6/614 | validated | - |
| *C16orf89* | 7870 | chr16:5115751 | rs142191897 | C>A | missense | 6/612 | validated | - |
| *C17orf66* | 37150 | chr17:34186056 | - | C>G | missense | 0/614 | - | validated |
| *C17orf66* | 17678 | chr17:34192351 | rs141724302 | G>A | missense | 6/614 | validated | validated |
| *C17orf70* | 37994 | chr17:79517729 | rs62076033 | G>A | missense | 1/610 | validated | - |
| *C19orf22* | 7531 | chr19:900951 | rs78580402 | A>G | missense | 5/598 | validated | - |
| *C19orf57* | 37674 | chr19:13993709 | rs111386677 | C>T | missense | 2/614 | validated | - |
| *C1orf168* | 3836, 7936 | chr1:57254697 | rs41305876 | G>A | missense | 5/616 | validated | validated |
| *C1orf27* | 7936 | chr1:186368092 | rs76602544 | A>C | missense | 10/588 | validated | - |
| *C1QTNF6* | 17342 | chr22:37578388 | rs17812681 | C>T | missense | 7/612 | validated | - |
| *C20orf118* | 37994 | chr20:35521264 | rs138927042 | G>T | splicing | 3/614 | validated | validated |
| *C20orf165* | 7503 | chr20:44515322 | rs76638086 | C>T | missense | 1/614 | validated | - |
| *C20orf3* | 7936 | chr20:24952147 | rs78661674 | G>A | missense | 12/614 | validated | - |
| *C2CD3* | 37037 | chr11:73850002 | rs72984881 | G>C | missense | 10/614 | validated | - |
| *C2orf51* | 7936 | chr2:88828961 | rs145039164 | G>A | missense | 0/616 | validated | - |
| *C2orf85* | 18074 | chr2:242814639 | rs75447317 | T>C | missense | 5/610 | validated | - |
| *C2orf85* | 18074 | chr2:242815059 | rs28368764 | C>T | missense | 6/612 | validated | - |
| *C6orf57* | 17678 | chr6:71298323 | rs146446063 | C>T | missense | 15/614 | validated | - |
| *C7orf23* | 37037 | chr7:86827302 | rs111700668 | G>T | missense | 5/614 | validated | - |
| *C7orf26* | 17245 | chr7:6631481 | - | G>T | missense | 0/614 | - | validated |
| *C7orf57* | 7936 | chr7:48086151 | rs111480809 | G>A | missense | 14/610 | validated | - |
| *C9* | 37425 | chr5:39289043 | rs141645272 | A>G | missense | 0/614 | validated | - |
| *C9orf50* | 37674 | chr9:132375739 | rs41276772 | A>G | missense | 0/608 | validated | - |
| *CADPS2/RNF133* | 17342 | chr7:122338970 | rs71574716 | C>T | missense | 21/614 | validated | - |
| *CATSPER4* | 37232 | chr1:26517811 | rs142583631 | A>G | missense | 9/614 | validated | - |
| *CCDC141* | 7658 | chr2:179733939 | rs141939661 | C>T | missense | 4/616 | validated | - |
| *CCDC38* | 37994 | chr12:96272113 | rs149118920 | A>G | missense | 0/614 | - | validated |
| *CCDC74A* | 17351 | chr2:132287879 | - | G>A | missense | 0/598 | - | validated |
| *CCDC74A* | 37037 | chr2:132290912 | rs140033985 | G>A | missense | 12/614 | - | validated |
| *CCDC81* | 37150 | chr11:86126337 | rs35587986 | T>G | missense | 9/614 | validated | - |
| *CCIN* | 7590 | chr9:36169894 | rs117143733 | A>G | missense | 8/614 | validated | - |
| *CCL14* | 37232 | chr17:34313612 | rs75238886 | G>A | missense | 20/614 | validated | - |
| *CCR5* | 17122 | chr3:46414557 | rs1799863 | T>A | missense | 18/616 | validated | - |
| *CCRL2* | 17351 | chr3:46449863 | rs111863112 | G>A | missense | 11/614 | validated | - |
| *CD34* | 7713 | chr1:208073205 | rs35359362 | C>A | missense | 11/616 | validated | - |
| *CDAN1* | 7658 | chr15:43022806 | rs140014115 | G>A | missense | 1/614 | validated | - |
| *CDH1* | 7936 | chr16:68855966 | rs35187787 | G>A | missense | 4/614 | validated | - |
| *CDH11* | 17122 | chr16:65016165 | rs76181686 | C>G | missense | 16/614 | validated | validated |
| *CDH23* | 17122 | chr10:73537449 | rs41281330 | G>A | missense | 10/598 | validated | - |
| *CDH26* | 17122 | chr20:58559852 | rs150010707 | G>T | missense | 0/614 | validated | - |
| *CDH9* | 17351 | chr5:26885797 | rs34490509 | T>C | missense | 0/614 | validated | validated |
| *CDHR1* | 7590 | chr10:85972932 | rs137876961 | A>G | missense | 4/614 | validated | - |
| *CDKAL1* | 7872 | chr6:20546697 | rs111739077 | G>A | missense | 4/614 | validated | validated |
| *CENPE* | 17678 | chr4:104080217 | rs75568479 | A>C | missense | 0/614 | validated | - |
| *CEP250* | 37425 | chr20:34064340 | - | C>T | missense | 0/614 | validated | - |
| *CEP290* | 37425 | chr12:88472996 | rs61941020 | C>T | missense | 3/610 | validated | - |
| *CEP290* | 37117 | chr12:88508258 | rs79705698 | T>C | missense | 15/612 | validated | - |
| *CEP68* | 18074 | chr2:65299420 | rs35501092 | T>C | missense | 12/616 | validated | - |
| *CGN* | 17678 | chr1:151491836 | rs140720174 | C>T | missense | 1/616 | validated | - |
| *CGREF1* | 7713 | chr2:27324669 | rs61753362 | C>T | missense | 18/614 | validated | - |
| *CGRRF1* | 37425 | chr14:54997751 | rs34839928 | C>T | missense | 9/614 | validated | - |
| *CHTF18* | 18074 | chr16:845754 | - | C>T | missense | 0/518 | validated | - |
| *CHTOP* | 37150 | chr1:153615820 | rs74844193 | G>A | missense | 19/616 | validated | - |
| *CIC* | 37037 | chr19:42798982 | - | C>T | missense | 0/614 | - | validated |
| *CIT* | 7637 | chr12:120220431 | - | C>T | missense | 0/614 | - | validated |
| *CIZ1* | 7606 | chr9:130941377 | rs45554035 | T>C | missense | 0/610 | validated | - |
| *CLCN2* | 37994 | chr3:184076909 | rs151257924 | C>T | missense | 0/616 | validated | validated |
| *CLDN3* | 7435 | chr7:73183979 | rs139191328 | G>A | missense | 4/540 | validated | validated |
| *CLIP1* | 7590 | chr12:122825398 | rs61954403 | C>T | missense | 9/614 | validated | - |
| *CLYBL* | 17122 | chr13:100425097 | rs17577293 | G>T | missense | 30/614 | validated | - |
| *CNTN5* | 17342 | chr11:100168410 | rs141228828 | T>A | missense | 3/612 | validated | validated |
| *COL22A1* | 7623 | chr8:139815174 | rs141112517 | C>G | missense | 2/614 | validated | - |
| *COL4A4* | 7531 | chr2:227915847 | rs13027659 | C>T | missense | 13/616 | validated | - |
| *COL5A3* | 7590 | chr19:10085062 | rs111357806 | C>T | missense | 7/614 | validated | - |
| *COL6A3* | 18074 | chr2:238253016 | rs151079701 | G>A | missense | 0/616 | validated | - |
| *COL6A3* | 17478 | chr2:238271991 | rs146546544 | G>A | missense | 0/616 | - | validated |
| *COL6A6* | 37994 | chr3:130282383 | rs114511272 | C>A | missense | 11/612 | validated | - |
| *COMP* | 7637 | chr19:18897440 | rs61739916 | T>C | missense | 27/612 | validated | - |
| *CPZ* | 37232 | chr4:8613762 | rs147588134 | G>T | missense | 2/616 | validated | - |
| *CRISP2* | 7637 | chr6:49663567 | rs36069724 | A>G | missense | 16/614 | validated | - |
| *CSMD1* | 37117 | chr8:2965294 | - | G>C | missense | 2/614 | validated | validated |
| *CSMD1* | 17122 | chr8:3253832 | - | C>T | missense | 0/604 | - | validated |
| *CTSZ* | 17122 | chr20:57572709 | rs117245400 | A>G | missense | 7/614 | validated | - |
| *CUL7* | 37425 | chr6:43014022 | rs61732148 | G>A | missense | 16/614 | validated | - |
| *CXorf59* | 37117 | chr23:36091379 | rs74495781 | A>C | missense | 5/451 | validated | - |
| *CYP2C18* | 17302 | chr10:96447920 | rs117111102 | C>T | missense | 14/614 | validated | - |
| *CYP4B1* | 17545 | chr1:47280830 | rs45467195 | A>G | missense | 9/616 | validated | - |
| *CYP4X1* | 7797 | chr1:47498961 | rs116257861 | G>A | missense | 0/616 | validated | - |
| *CYTL1* | 7637 | chr4:5016883 | rs11722554 | G>A | missense | 19/616 | validated | - |
| *DAO* | 7663 | chr12:109293187 | rs143550642 | G>A | missense | 0/614 | validated | - |
| *DCBLD2* | 7658 | chr3:98600385 | rs9838238 | T>C | missense | 30/612 | validated | - |
| *DCHS1* | 18074 | chr11:6661600 | rs117368891 | G>T | missense | 8/610 | validated | - |
| *DCLRE1A* | 37425 | chr10:115602192 | rs11196530 | T>A | missense | 4/614 | validated | - |
| *DEF6* | 17351 | chr6:35289153 | rs6917127 | A>C | missense | 0/614 | validated | - |
| *DEFB128* | 7637 | chr20:168558 | rs74181522 | A>G | missense | 7/614 | validated | - |
| *DLGAP2* | 37117 | chr8:1624708 | - | G>C | missense | 0/594 | - | validated |
| *DMXL1* | 17678 | chr5:118505962 | rs139856633 | C>T | missense | 1/614 | validated | - |
| *DMXL1* | 37674 | chr5:118506652 | - | C>T | missense | 0/614 | - | validated |
| *DNAAF1* | 17351 | chr16:84211403 | rs4150187 | C>G | missense | 0/614 | validated | - |
| *DNAH10* | 7936 | chr12:124356105 | - | G>T | missense | 0/610 | - | validated |
| *DNAH10* | 7936 | chr12:124401027 | rs61745785 | C>A | missense | 5/608 | validated | - |
| *DNAH6* | 7936 | chr2:84928399 | - | C>A | missense | 1/612 | validated | - |
| *DNAH8* | 37425 | chr6:38957948 | rs115630842 | C>T | missense | 0/614 | validated | - |
| *DNAH9* | 7590 | chr17:11840819 | rs144547132 | G>T | missense | 0/614 | validated | - |
| *DNAJC30* | 7506, 17351 | chr7:73097713 | rs61751896 | G>A | missense | 25/612 | validated | - |
| *DOCK8* | 7590 | chr9:312134 | rs11789099 | G>A | missense | 17/612 | validated | - |
| *DSEL* | 7503 | chr18:65179657 | rs12953840 | T>C | missense | 3/614 | validated | - |
| *DTHD1* | 37232 | chr4:36340755 | rs56412718 | C>A | missense | 29/614 | validated | - |
| *EDC4* | 7531 | chr16:67913976 | rs142731612 | G>A | missense | 0/608 | validated | - |
| *EFCAB5* | 17678 | chr17:28268857 | - | G>A | splicing | 2/610 | validated | - |
| *EFCAB6* | 37024 | chr22:44004465 | rs55698170 | C>T | missense | 4/614 | validated | - |
| *EFCAB7* | 37425 | chr1:64011657 | rs41313264 | A>G | missense | 4/616 | validated | - |
| *EFS* | 37425 | chr14:23829490 | rs2231801 | C>T | missense | 11/614 | validated | - |
| *EGFLAM* | 17351 | chr5:38418311 | rs146754357 | C>A | missense | 1/614 | validated | - |
| *EHHADH* | 37037 | chr3:184910078 | rs55752621 | G>A | missense | 8/616 | validated | - |
| *EHMT2* | 7435 | chr6:31864538 | rs115884658 | G>A | missense | 14/612 | validated | - |
| *EMILIN1* | 17342 | chr2:27308159 | rs36045790 | G>A | missense | 10/614 | validated | - |
| *EPHA1* | 17545 | chr7:143095153 | rs11768549 | C>T | missense | 12/612 | validated | - |
| *EPPK1* | 7745 | chr8:144941198 | rs79860671 | T>G | missense | 9/582 | validated | - |
| *ESPL1* | 17342 | chr12:53682043 | rs61737629 | C>G | missense | 8/608 | validated | - |
| *EVI2A* | 37150 | chr17:29645473 | rs140933050 | C>T | missense | 2/614 | validated | - |
| *F13A1* | 37994 | chr6:6251120 | rs3024477 | T>A | missense | 20/614 | validated | validated |
| *FAM166B* | 7590 | chr9:35563361 | rs75679360 | G>A | missense | 22/584 | validated | - |
| *FAM169A* | 37117 | chr5:74101028 | - | C>T | missense | 0/614 | - | validated |
| *FAM173B* | 7936 | chr5:10236693 | rs17360625 | A>G | missense | 14/612 | validated | - |
| *FAM184B* | 17545 | chr4:17690072 | rs61741063 | T>C | missense | 12/608 | validated | - |
| *FAM71A* | 37534 | chr1:212799139 | rs146657378 | A>G | missense | 8/616 | validated | - |
| *FANCD2* | 37150 | chr3:10115047 | - | G>A | splicing | 0/616 | validated | - |
| *FAT1* | 37037 | chr4:187518041 | rs72716244 | T>C | missense | 11/610 | validated | - |
| *FAT1* | 17545 | chr4:187549364 | rs111886222 | G>A | missense | 5/614 | validated | - |
| *FAT1* | 17545 | chr4:187557908 | rs113970444 | C>T | missense | 5/608 | validated | - |
| *FAT4* | 7713 | chr4:126373570 | rs75380987 | C>T | missense | 19/614 | validated | - |
| *FBF1* | 17545 | chr17:73915803 | rs113062332 | G>A | missense | 16/592 | validated | - |
| *FBN3* | 7590 | chr19:8175770 | rs17160194 | T>A | missense | 0/614 | validated | - |
| *FBN3* | 37674 | chr19:8183871 | rs35579498 | G>A | missense | 25/614 | validated | - |
| *FBP2* | 17342 | chr9:97321404 | rs72743247 | A>G | missense | 4/614 | validated | - |
| *FBXL14* | 17122 | chr12:1702929 | rs117331652 | T>G | missense | 5/604 | validated | - |
| *FBXO30* | 7658 | chr6:146121343 | - | C>G | missense | 0/614 | - | validated |
| *FBXO30* | 37994 | chr6:146126163 | rs150645956 | G>A | missense | 1/614 | validated | validated |
| *FBXO36* | 37425 | chr2:230841011 | rs61753284 | C>T | missense | 7/616 | validated | - |
| *FBXO40* | 37425 | chr3:121340955 | rs148776238 | G>A | missense | 12/616 | validated | - |
| *FGD6* | 37994 | chr12:95604078 | - | G>A | nonsense | 0/614 | - | validated |
| *FLII* | 7590 | chr17:18148534 | rs8821 | C>T | missense | 17/608 | validated | - |
| *FLJ43860* | 37037 | chr8:142500315 | - | G>A | missense | 9/570 | validated | - |
| *FRK* | 37674 | chr6:116325108 | rs34064900 | G>A | missense | 6/614 | validated | - |
| *FRY* | 17678 | chr13:32785102 | - | G>A | missense | 0/612 | validated | - |
| *FSIP2* | 7606 | chr2:186654867 | rs79762465 | G>C | missense | 23/612 | validated | - |
| *FSTL5* | 17302 | chr4:162697058 | rs72689202 | C>T | missense | 14/614 | validated | validated |
| *FYCO1* | 17351 | chr3:46003735 | rs41289620 | C>T | missense | 5/616 | validated | validated |
| *FYCO1* | 17351 | chr3:46008983 | rs149507450 | G>A | missense | 5/616 | validated | validated |
| *GAB2* | 37994 | chr11:77961261 | - | C>T | missense | 0/614 | - | validated |
| *GALM* | 17342 | chr2:38956836 | rs139320905 | C>G | missense | 0/616 | validated | validated |
| *GDF15* | 7506 | chr19:18499119 | rs146900068 | C>G | missense | 0/570 | validated | - |
| *GEMIN5* | 17678 | chr5:154307070 | rs35522740 | G>C | missense | 11/614 | validated | - |
| *GGT6* | 18074 | chr17:4463713 | rs62066362 | A>G | missense | 13/614 | validated | - |
| *GHRHR* | 7870 | chr7:31018852 | rs2228078 | T>C | missense | 13/612 | validated | - |
| *GJA8* | 7663, 37232 | chr1:147380740 | rs138140155 | A>G | missense | 6/616 | validated | - |
| *GLIS3* | 37425 | chr9:4118634 | rs143051164 | G>C | missense | 1/614 | validated | - |
| *GLUD2* | 37150 | chr23:120181950 | rs140532390 | C>T | missense | 0/451 | - | validated |
| *GPR113* | 17342 | chr2:26536727 | rs74987785 | C>T | missense | 9/594 | validated | - |
| *GPR142* | 7713 | chr17:72366761 | rs140763121 | G>A | missense | 2/610 | validated | - |
| *GPR84* | 17342 | chr12:54757199 | rs138447533 | A>G | missense | 0/614 | validated | validated |
| *GPRC6A* | 7590 | chr6:117113315 | rs41290852 | G>A | missense | 16/614 | validated | - |
| *GRIN3B* | 37024 | chr19:1008645 | rs78914045 | C>A | missense | 5/600 | validated | - |
| *GRM2* | 7658 | chr3:51747288 | rs116567227 | G>A | missense | 9/612 | validated | - |
| *GRM6* | 37037 | chr5:178413163 | rs62638623 | G>C | missense | 7/614 | validated | - |
| *GUCA1B* | 7936 | chr6:42153428 | rs139923590 | C>A | missense | 5/614 | validated | - |
| *HEATR2* | 17122 | chr7:803506 | rs73258248 | C>T | missense | 4/614 | validated | - |
| *HEATR7B1* | 37037 | chr2:234713681 | rs79208193 | G>T | missense | 23/592 | - | validated |
| *HHIPL2* | 7713 | chr1:222712108 | rs116359984 | G>T | missense | 3/616 | validated | - |
| *HIF1A* | 17545 | chr14:62204808 | rs41508050 | C>T | missense | 1/614 | validated | - |
| *HIVEP1* | 37024 | chr6:12163802 | rs74910145 | G>A | missense | 0/614 | validated | - |
| *HPS6* | 7435 | chr10:103825865 | - | G>A | missense | 0/612 | - | validated |
| *HTR7* | 17302 | chr10:92509055 | rs114969659 | G>A | missense | 0/614 | validated | validated |
| *IGDCC4* | 7658 | chr15:65677446 | rs116928937 | C>A | missense | 9/550 | validated | - |
| *IGSF10* | 37994 | chr3:151154509 | rs112889898 | C>T | missense | 7/616 | validated | - |
| *IL17RB* | 17545 | chr3:53889368 | rs2232337 | G>A | missense | 6/616 | validated | - |
| *IL4R* | 7506 | chr16:27374696 | rs3024678 | C>T | missense | 13/612 | validated | - |
| *INADL* | 17122 | chr1:62582847 | rs41289430 | C>G | missense | 17/614 | validated | - |
| *IQCB1* | 7658 | chr3:121500697 | rs11920543 | G>A | missense | 9/616 | validated | - |
| *IQGAP2* | 7637 | chr5:75960968 | rs34968964 | G>C | missense | 4/614 | validated | - |
| *IRAK2* | 37425 | chr3:10219567 | rs11465864 | C>A | missense | 14/616 | validated | - |
| *ISM1* | 37425 | chr20:13251316 | rs77255807 | T>C | missense | 18/612 | validated | - |
| *ITGA9* | 17342 | chr3:37785454 | rs140716372 | G>A | missense | 5/616 | validated | - |
| *ITSN2* | 7435 | chr2:24435599 | rs41281481 | G>A | missense | 30/616 | validated | - |
| *JAK3* | 37150 | chr19:17945696 | rs3213409 | C>T | missense | 7/614 | validated | - |
| *JARID2* | 37994 | chr6:15496930 | rs150448457 | C>T | missense | 6/602 | validated | validated |
| *KCNB1* | 7503 | chr20:47989527 | rs34280195 | C>T | missense | 7/612 | validated | - |
| *KCNB2* | 3836 | chr8:73849256 | rs144823279 | C>T | missense | 0/614 | - | validated |
| *KCNB2* | 17678 | chr8:73850175 | - | A>G | missense | 0/614 | - | validated |
| *KEL* | 17122 | chr7:142655008 | rs8176058 | G>A | missense | 29/614 | validated | - |
| *KIAA1257* | 37425 | chr3:128711946 | rs115301439 | C>A | missense | 0/588 | validated | - |
| *KIAA1468* | 7663 | chr18:59854886 | rs146488728 | G>A | missense | 6/610 | validated | - |
| *KIAA1614* | 7713, 37232 | chr1:180905263 | rs17302207 | C>T | missense | 23/614 | validated | - |
| *KIAA1949* | 37425 | chr6:30652729 | rs2213944 | G>A | missense | 3/612 | validated | - |
| *KLHL1* | 17342 | chr13:70370894 | - | T>C | missense | 0/614 | - | validated |
| *KNG1* | 17545 | chr3:186461524 | rs76438938 | C>T | nonsense | 19/616 | validated | - |
| *LAMA2* | 17342 | chr6:129513850 | rs118083923 | T>A | missense | 1/614 | validated | - |
| *LARS2* | 17545 | chr3:45518073 | rs71645922 | C>A | missense | 24/616 | validated | - |
| *LCN12* | 37674 | chr9:139849847 | rs115310708 | G>C | missense | 0/610 | validated | - |
| *LCN2* | 7606 | chr9:130914200 | rs79993583 | C>T | missense | 0/614 | validated | - |
| *LDLRAD3* | 7797 | chr11:36057670 | rs144816501 | G>A | missense | 1/612 | validated | - |
| *LIG1* | 37037 | chr19:48624555 | rs146309259 | C>T | missense | 3/596 | validated | - |
| *LIPI* | 18074 | chr21:15524921 | rs74369337 | C>T | missense | 17/614 | validated | - |
| *LIPT2* | 17678 | chr11:74203256 | rs141813641 | T>A | missense | 0/600 | validated | - |
| *LLGL1* | 17678 | chr17:18145552 | rs149387516 | C>A | missense | 0/614 | - | validated |
| *LRPAP1* | 7637 | chr4:3526715 | rs146399188 | C>T | missense | 0/616 | validated | validated |
| *LRRC8E* | 7506 | chr19:7964482 | rs45584934 | G>A | missense | 0/614 | validated | - |
| *LSM14A* | 7531 | chr19:34706203 | rs36006556 | G>A | missense | 23/614 | validated | - |
| *LTBP2* | 37994 | chr14:74976452 | rs61505039 | C>T | missense | 1/612 | validated | - |
| *LYSMD3* | 17342 | chr5:89815108 | rs62375061 | G>T | missense | 14/612 | validated | - |
| *MAP3K1* | 7637 | chr5:56155672 | rs56069227 | A>G | missense | 15/614 | validated | - |
| *MAP3K10* | 7506 | chr19:40698441 | rs36102209 | C>A | missense | 1/602 | validated | - |
| *MAP3K4* | 17342 | chr6:161470544 | rs143540599 | T>G | missense | 0/614 | validated | - |
| *MAP3K4* | 17342 | chr6:161508880 | rs35533223 | A>C | missense | 22/614 | validated | - |
| *MCM7* | 7797 | chr7:99695873 | - | G>A | missense | 0/614 | validated | - |
| *MDC1* | 37425 | chr6:30679963 | rs2844707 | A>C | missense | 3/612 | validated | - |
| *MDC1* | 37425 | chr6:30680968 | rs2517560 | C>T | missense | 2/612 | validated | - |
| *MDM1* | 37232 | chr12:68720534 | rs117673673 | A>G | missense | 6/614 | validated | - |
| *MDP1/NEDD8-MDP1* | 7531, 37674 | chr14:24683304 | rs145254894 | C>A | missense | 9/614 | validated | - |
| *MELK* | 7590 | chr9:36583642 | rs114617403 | A>G | missense | 6/614 | validated | - |
| *MGA* | 37232 | chr15:42021512 | rs17677811 | T>C | missense | 21/612 | validated | - |
| *MGST3* | 37150 | chr1:165619079 | rs6681 | C>T | missense | 27/616 | validated | - |
| *MICA* | 37425 | chr6:31378965 | rs1051791 | G>A | missense | 5/614 | validated | - |
| *MLL4* | 17342 | chr19:36216691 | - | C>T | missense | 0/562 | - | validated |
| *MLL5* | 37037 | chr7:104747899 | rs117986340 | G>T | missense | 23/614 | validated | - |
| *MNS1* | 37425 | chr15:56723692 | rs144217705 | C>T | missense | 1/614 | validated | - |
| *MOG* | 7936 | chr6:29627222 | rs138169338 | C>A | missense | 3/612 | validated | - |
| *MRGPRX4* | 18074 | chr11:18195609 | rs146132319 | C>T | missense | 5/614 | validated | - |
| *MRPL3* | 7713 | chr3:131188574 | rs2291381 | A>G | missense | 7/616 | validated | - |
| *MTUS1* | 7663 | chr8:17612686 | rs61733697 | A>C | missense | 11/614 | validated | - |
| *MUC17* | 37024 | chr7:100692149 | rs117698587 | C>T | nonsense | 0/614 | validated | - |
| *MYH8* | 7590 | chr17:10298599 | rs139344968 | C>T | missense | 1/614 | validated | validated |
| *MYH8* | 7590 | chr17:10298628 | rs145711576 | C>G | missense | 1/614 | validated | validated |
| *MYO1A* | 17678 | chr12:57431366 | rs148808080 | C>T | missense | 0/614 | validated | - |
| *MYO1G* | 37674 | chr7:45009432 | - | G>A | missense | 0/612 | validated | - |
| *MYO3A* | 17122 | chr10:26463052 | rs35575696 | C>A | missense | 6/614 | validated | - |
| *MYO9A* | 17122, 17302 | chr15:72189952 | rs80283650 | T>A | missense | 13/614 | validated | validated |
| *MYOM1* | 37232 | chr18:3135575 | rs115382168 | T>C | missense | 0/598 | validated | - |
| *MYOM1* | 17351 | chr18:3135644 | rs149528866 | C>T | missense | 3/604 | validated | validated |
| *MYOM1* | 17351 | chr18:3215083 | - | T>C | missense | 1/588 | validated | validated |
| *MYOM2* | 7663 | chr8:2050520 | - | G>A | missense | 1/614 | validated | - |
| *NARF* | 7590 | chr17:80439004 | rs113870312 | A>G | missense | 4/614 | validated | - |
| *NEB* | 37674 | chr2:152468776 | rs35740585 | T>C | missense | 12/606 | validated | - |
| *NEBL* | 17342 | chr10:21158716 | - | G>A | nonsense | 0/614 | - | validated |
| *NFX1* | 37150 | chr9:33366682 | rs146508853 | G>A | missense | 14/614 | validated | - |
| *NINL* | 17122, 37425 | chr20:25439036 | rs41310175 | G>A | missense | 21/614 | - | validated |
| *NIPAL1* | 7531 | chr4:48038163 | rs142281486 | A>G | missense | 0/614 | validated | - |
| *NLRP5* | 17351 | chr19:56515319 | - | A>C | missense | 1/606 | validated | - |
| *NMB* | 17678 | chr15:85198631 | - | G>A | missense | 0/614 | validated | - |
| *NOL8* | 17342 | chr9:95077470 | rs61742574 | T>A | missense | 26/610 | validated | - |
| *NPC1L1* | 17351 | chr7:44561344 | rs149782907 | G>A | missense | 0/614 | validated | - |
| *NPHP4* | 17122 | chr1:5965455 | rs571655 | C>T | missense | 5/600 | validated | - |
| *NRCAM* | 17342 | chr7:107822355 | rs150373689 | G>A | missense | 1/614 | validated | validated |
| *NRXN2* | 17478 | chr11:64453180 | rs146308270 | C>T | missense | 1/614 | validated | validated |
| *NTSR2* | 37994 | chr2:11802293 | - | A>T | missense | 0/616 | - | validated |
| *NUP85* | 37994 | chr17:73221527 | rs61760968 | G>A | missense | 5/614 | validated | - |
| *OAS3* | 7936, 17545 | chr12:113403675 | rs61942233 | C>T | nonsense | 11/602 | validated | - |
| *ODZ2* | 7590 | chr5:167474474 | rs114898031 | A>G | missense | 14/600 | validated | - |
| *OFCC1* | 7606 | chr6:9809860 | - | C>T | missense | 0/602 | - | validated |
| *OFCC1* | 7606 | chr6:9900660 | rs148761621 | C>A | nonsense | 0/614 | - | validated |
| *OGFR* | 17122 | chr20:61444939 | rs11543349 | G>C | missense | 15/610 | validated | - |
| *OPTC* | 17351 | chr1:203466190 | rs61731863 | C>T | missense | 0/616 | validated | - |
| *OR10R2* | 18074 | chr1:158450142 | rs117155388 | C>T | nonsense | 0/616 | validated | - |
| *OR1A1* | 37674 | chr17:3119648 | rs62090945 | C>T | missense | 0/614 | validated | - |
| *OR2AT4* | 37994 | chr11:74800417 | rs145245435 | A>C | missense | 1/614 | validated | - |
| *OR2M4* | 7936 | chr1:248403015 | rs41303133 | C>A | missense | 6/616 | validated | - |
| *OR4A16* | 17478 | chr11:55111500 | rs117538213 | T>C | missense | 30/614 | validated | - |
| *OR4C16* | 37037 | chr11:55340387 | rs141913559 | G>A | missense | 0/614 | validated | - |
| *OR4M2* | 7435 | chr15:22368588 | rs111484216 | A>G | missense | 8/614 | validated | - |
| *OR51E1* | 37674 | chr11:4674652 | rs149616162 | A>G | missense | 3/614 | validated | - |
| *OR51I2* | 7435 | chr11:5475505 | rs75620804 | C>T | missense | 16/614 | validated | - |
| *OR51L1* | 7435, 37674 | chr11:5020933 | rs61729748 | A>T | missense | 15/614 | validated | - |
| *OR52K2* | 7531 | chr11:4471469 | rs61997232 | A>C | missense | 18/614 | validated | - |
| *OR52N2* | 37674 | chr11:5842283 | rs61875922 | T>A | missense | 2/614 | validated | - |
| *OTOF* | 17342 | chr2:26703734 | rs55676840 | C>T | missense | 4/610 | validated | - |
| *OTOF* | 17545 | chr2:26750775 | rs150132765 | G>A | missense | 1/616 | validated | - |
| *PADI3* | 17122 | chr1:17596817 | rs145296609 | C>T | missense | 3/616 | validated | - |
| *PBLD* | 37534 | chr10:70045105 | - | C>T | missense | 0/614 | validated | - |
| *PDCD11* | 7435 | chr10:105202091 | rs146747336 | G>T | missense | 2/614 | validated | validated |
| *PEG3* | 37425 | chr19:57325545 | rs56237501 | G>C | missense | 9/614 | validated | - |
| *PEX5* | 37425 | chr12:7362713 | rs146567534 | G>A | missense | 2/614 | validated | - |
| *PEX6* | 37425 | chr6:42932835 | rs2274516 | C>T | missense | 18/614 | validated | - |
| *PHC3* | 17122 | chr3:169831268 | rs73181210 | T>C | missense | 2/612 | validated | - |
| *PHLDB3* | 37037 | chr19:43991217 | rs138387298 | C>T | missense | 4/584 | validated | - |
| *PKHD1* | 7637 | chr6:51893107 | rs41273726 | T>C | missense | 7/614 | validated | - |
| *PLCD1* | 37994 | chr3:38051211 | rs75495843 | G>A | missense | 14/614 | - | validated |
| *PLEKHM3* | 7713 | chr2:208842276 | rs115156379 | C>G | missense | 17/616 | validated | - |
| *PLK4* | 17342 | chr4:128817999 | - | A>T | missense | 0/614 | - | validated |
| *POLE* | 7637 | chr12:133240613 | - | C>T | missense | 0/614 | validated | validated |
| *POLE* | 17678 | chr12:133252760 | - | A>C | missense | 0/614 | - | validated |
| *POLG2* | 18074 | chr17:62479069 | rs61751983 | A>C | missense | 0/614 | validated | - |
| *POLH* | 7936 | chr6:43582176 | rs151095678 | C>A | missense | 1/614 | validated | - |
| *POLM* | 7506, 17351 | chr7:44118394 | rs28382644 | C>G | missense | 6/614 | validated | validated |
| *POLN* | 7637 | chr4:2195009 | rs11725880 | G>A | missense | 11/616 | validated | - |
| *POLR3E* | 7531 | chr16:22339847 | rs148649380 | C>T | missense | 0/612 | validated | - |
| *POP4* | 7531 | chr19:30104871 | rs61731483 | G>T | missense | 18/614 | validated | - |
| *POU4F2* | 7797 | chr4:147561147 | rs147517729 | C>A | missense | 8/614 | validated | - |
| *PPIP5K2* | 37674 | chr5:102537298 | rs17155147 | C>T | missense | 24/614 | validated | - |
| *PPM1J* | 17678 | chr1:113254648 | rs78366259 | A>G | missense | 12/616 | validated | - |
| *PPP1R15A* | 7506 | chr19:49376683 | rs45533432 | A>G | missense | 2/614 | validated | - |
| *PPP3R2* | 37425 | chr9:104357062 | rs151333989 | C>T | missense | 3/614 | validated | - |
| *PPRC1* | 37425 | chr10:103907055 | rs34169738 | C>T | missense | 8/614 | validated | - |
| *PRDM6* | 17478 | chr5:122495236 | - | G>A | missense | 0/612 | - | validated |
| *PRICKLE1* | 37232 | chr12:42862463 | rs61924369 | C>T | missense | 0/614 | - | validated |
| *PRICKLE1* | 37232 | chr12:42864125 | - | C>G | missense | 0/614 | - | validated |
| *PRSS38* | 37534 | chr1:228004966 | rs61826983 | C>A | missense | 16/616 | validated | - |
| *PRUNE2* | 7590 | chr9:79465455 | rs41304230 | C>T | missense | 6/614 | validated | - |
| *PTCH2* | 17545 | chr1:45295296 | rs139624405 | C>T | missense | 6/616 | validated | - |
| *PTGFRN* | 37425 | chr1:117503940 | rs34625170 | G>A | missense | 20/616 | validated | - |
| *PTPRC* | 7713 | chr1:198668761 | rs41269905 | G>C | missense | 9/616 | validated | - |
| *PUS3* | 17122 | chr11:125765528 | rs35681508 | G>A | missense | 27/614 | validated | - |
| *PXDNL* | 37674 | chr8:52284560 | rs117752382 | A>T | nonsense | 15/598 | validated | - |
| *RAB3GAP2* | 7531 | chr1:220379266 | - | C>T | missense | 0/616 | validated | - |
| *RAD9B* | 37232 | chr12:110952892 | rs61758787 | C>G | missense | 1/612 | validated | - |
| *RASSF6* | 17545 | chr4:74447930 | rs72649118 | T>C | missense | 10/614 | validated | - |
| *RBM46* | 7531 | chr4:155719189 | rs79167802 | T>G | missense | 7/614 | validated | - |
| *RDH11* | 17122, 37037 | chr14:68159269 | rs80140987 | C>T | missense | 11/614 | validated | validated |
| *RELN* | 17342 | chr7:103234202 | rs55689103 | C>T | missense | 20/614 | validated | - |
| *REPS1* | 17122 | chr6:139265115 | - | G>A | missense | 0/614 | - | validated |
| *RGNEF* | 7663 | chr5:73153552 | rs115243197 | G>A | missense | 6/610 | validated | - |
| *RHOD* | 17478 | chr11:66837996 | rs34270544 | G>A | missense | 12/614 | validated | - |
| *ROGDI* | 7870 | chr16:4847824 | rs138409264 | C>T | missense | 4/610 | validated | - |
| *RPL22L1* | 37994 | chr3:170584249 | rs13462 | C>A | missense | 5/600 | validated | - |
| *RRP7A* | 17122 | chr22:42914033 | rs146723886 | T>C | missense | 8/614 | validated | - |
| *RSL1D1* | 37674 | chr16:11933743 | rs34999527 | C>A | missense | 6/614 | validated | - |
| *RTN4IP1* | 7590 | chr6:107040160 | rs138679791 | C>A | missense | 0/614 | validated | - |
| *SAC3D1* | 17545 | chr11:64811900 | - | C>T | missense | 6/598 | validated | - |
| *SAMD10* | 37674 | chr20:62608720 | rs145097158 | T>G | missense | 7/594 | validated | - |
| *SAMD9* | 37037 | chr7:92734051 | rs117649834 | C>T | missense | 29/614 | validated | - |
| *SASH1* | 37994 | chr6:148865690 | rs140316955 | C>A | missense | 3/604 | - | validated |
| *SCAF11* | 37150 | chr12:46320311 | rs114180943 | G>T | missense | 0/614 | validated | - |
| *SCUBE3* | 17351 | chr6:35212504 | rs139323019 | G>A | missense | 0/614 | validated | - |
| *SEC24D* | 7713 | chr4:119673889 | rs141180741 | G>A | missense | 4/614 | validated | - |
| *SELV* | 37037 | chr19:40006628 | rs145756298 | T>C | missense | 2/600 | validated | - |
| *SEPT9* | 7713 | chr17:75398290 | - | C>T | missense | 0/608 | validated | - |
| *SEZ6* | 37150 | chr17:27308971 | - | G>T | missense | 5/590 | validated | - |
| *SGK223* | 17545 | chr8:8185746 | rs56215812 | T>C | missense | 10/612 | validated | - |
| *SH2D7* | 7658 | chr15:78393352 | - | C>T | missense | 1/608 | validated | - |
| *SH3BP5* | 7637 | chr3:15297787 | rs116459865 | T>C | missense | 6/616 | validated | - |
| *SIGLEC12* | 7531 | chr19:52004878 | rs148809065 | C>T | missense | 13/614 | validated | - |
| *SKIV2L* | 17351 | chr6:31929737 | rs36038685 | C>T | missense | 11/612 | validated | - |
| *SLC10A2* | 17122 | chr13:103701690 | rs56398830 | G>A | missense | 7/614 | validated | validated |
| *SLC26A2* | 37994 | chr5:149360630 | rs78676079 | C>T | missense | 17/614 | validated | validated |
| *SLC26A3* | 37037 | chr7:107427322 | rs34407351 | A>C | missense | 26/614 | validated | - |
| *SLC26A3* | 17122 | chr7:107434192 | - | A>T | missense | 0/614 | - | validated |
| *SLC2A9* | 17545 | chr4:9922187 | rs112404957 | G>A | missense | 8/616 | validated | - |
| *SLC36A2* | 17678 | chr5:150723155 | rs77010315 | C>A | missense | 8/614 | validated | - |
| *SLC6A17* | 17678 | chr1:110740129 | rs143189177 | G>A | missense | 1/616 | validated | - |
| *SLC6A20* | 7658 | chr3:45801393 | rs61731475 | T>C | missense | 5/616 | validated | - |
| *SLC8A3* | 37425 | chr14:70633411 | rs41286548 | C>T | missense | 13/614 | validated | - |
| *SLFN13* | 37425 | chr17:33771996 | rs72483216 | G>A | missense | 21/614 | validated | - |
| *SLIT3* | 17342, 18074 | chr5:168180047 | rs34260167 | C>T | missense | 7/614 | validated | validated |
| *SMARCC1* | 17545 | chr3:47777540 | - | A>G | missense | 0/616 | validated | - |
| *SOHLH1* | 37037 | chr9:138586263 | rs144035874 | G>T | missense | 3/610 | validated | - |
| *SORBS1* | 37425 | chr10:97194397 | rs138975797 | C>G | missense | 0/614 | validated | - |
| *SORBS2* | 7936 | chr4:186583330 | rs61734841 | A>G | missense | 6/614 | validated | - |
| *SOS2* | 37024 | chr14:50655307 | rs61755579 | C>T | missense | 13/614 | - | validated |
| *SOS2* | 37024 | chr14:50655357 | rs72681869 | G>C | missense | 6/614 | validated | validated |
| *SOS2* | 37425 | chr14:50655380 | rs137961578 | C>G | missense | 0/614 | validated | - |
| *SP100* | 37425 | chr2:231326031 | rs116484154 | G>A | missense | 4/616 | validated | - |
| *SPATA12* | 17351, 17545 | chr3:57108101 | rs76587478 | C>T | missense | 9/616 | validated | - |
| *SPECC1L* | 17478 | chr22:24717510 | rs56168869 | C>T | missense | 7/614 | validated | - |
| *SPNS3* | 37150 | chr17:4349389 | rs147085241 | C>T | missense | 0/614 | - | validated |
| *SPNS3* | 7658 | chr17:4391185 | rs149163440 | C>G | missense | 3/614 | - | validated |
| *SREBF2* | 17478 | chr22:42271378 | - | A>C | missense | 0/614 | - | validated |
| *SSH3* | 17478 | chr11:67074393 | rs61731165 | G>A | missense | 15/614 | validated | - |
| *ST3GAL5* | 37150 | chr2:86067277 | - | C>A | missense | 0/616 | validated | - |
| *STAB2* | 37994 | chr12:104138980 | rs116894406 | G>A | missense | 2/614 | validated | - |
| *STXBP5* | 37994 | chr6:147635108 | rs144099092 | C>G | missense | 2/614 | validated | validated |
| *STXBP5* | 7623 | chr6:147636753 | rs148830578 | A>G | missense | 2/614 | validated | validated |
| *SYN1* | 37674 | chr23:47466545 | - | C>T | missense | 0/451 | - | validated |
| *SYNE1* | 17122 | chr6:152779933 | rs34610829 | G>A | missense | 14/614 | validated | - |
| *SYNE2* | 37994 | chr14:64676751 | rs36215895 | C>T | missense | 1/612 | - | validated |
| *TAF3* | 7506 | chr10:8006519 | rs17366712 | G>C | missense | 22/612 | validated | - |
| *TAS2R8* | 7658 | chr12:10959438 | rs74611066 | G>A | missense | 6/614 | validated | - |
| *TAS2R9* | 7658 | chr12:10961978 | rs77609577 | C>T | missense | 6/614 | validated | - |
| *TBC1D2B* | 17122, 17302 | chr15:78337303 | rs61732053 | C>T | missense | 25/608 | validated | - |
| *TCAP* | 18074 | chr17:37822174 | rs45578741 | C>T | missense | 3/588 | validated | - |
| *TET2* | 7531, 7713 | chr4:106156163 | rs61744960 | G>A | missense | 29/614 | validated | - |
| *TGM1* | 37425 | chr14:24724663 | rs35312232 | C>T | missense | 11/612 | validated | - |
| *TMC1* | 37425 | chr9:75355093 | rs11143384 | C>T | missense | 1/614 | validated | - |
| *TMC5* | 37024 | chr16:19475099 | rs61737709 | C>A | missense | 23/614 | validated | - |
| *TMC7* | 37534 | chr16:19020725 | rs118019760 | A>C | missense | 4/614 | validated | - |
| *TMC7* | 37232 | chr16:19033065 | - | C>T | missense | 0/614 | - | validated |
| *TMCO4* | 18074 | chr1:20072082 | rs35760408 | C>T | missense | 17/616 | validated | - |
| *TMCO7* | 17678 | chr16:68936305 | - | G>T | missense | 0/612 | validated | - |
| *TMEM155* | 17545 | chr4:122682720 | rs138330999 | C>T | missense | 9/614 | validated | - |
| *TMEM176B* | 17122 | chr7:150493495 | rs11546671 | G>A | missense | 27/614 | validated | - |
| *TMEM184A* | 17545 | chr7:1587440 | rs61747419 | G>A | missense | 6/584 | validated | - |
| *TMEM70* | 17678 | chr8:74893653 | rs77410280 | G>A | missense | 12/614 | validated | - |
| *TNFRSF10C* | 7531 | chr8:22974278 | rs76324416 | C>T | missense | 13/614 | validated | - |
| *TNFRSF8* | 37037 | chr1:12175658 | rs2230624 | G>A | missense | 6/616 | validated | - |
| *TNRC6A* | 7936 | chr16:24815534 | rs146427035 | A>G | missense | 4/614 | validated | - |
| *TNXB* | 18074 | chr6:32049895 | rs138300355 | G>T | missense | 0/592 | validated | - |
| *TOPORS* | 37232 | chr9:32542278 | rs17857515 | T>C | missense | 5/614 | validated | - |
| *TP63* | 17302 | chr3:189607152 | rs148076109 | C>A | missense | 4/616 | validated | validated |
| *TPMT* | 7713, 39312 | chr6:18139228 | rs1800460 | C>T | missense | 23/614 | validated | - |
| *TRAFD1* | 37232 | chr12:112583447 | rs79680080 | A>C | missense | 7/614 | validated | - |
| *TRIM55* | 17678 | chr8:67047224 | rs141724668 | G>A | splicing | 0/614 | validated | validated |
| *TRIM55* | 37425 | chr8:67067937 | - | C>T | missense | 1/614 | - | validated |
| *TRIP11* | 7590 | chr14:92465749 | rs35007347 | C>T | missense | 12/614 | validated | - |
| *TRPM6* | 7590 | chr9:77367203 | rs55679040 | T>C | missense | 10/614 | validated | - |
| *TRPM6* | 7506 | chr9:77454973 | rs150874152 | C>T | missense | 1/614 | validated | validated |
| *TSEN54* | 37994 | chr17:73520380 | rs144662042 | C>T | missense | 5/614 | validated | validated |
| *TSPAN10* | 37425 | chr17:79612633 | rs34379910 | T>C | missense | 15/588 | validated | - |
| *TSPYL5* | 37425 | chr8:98289238 | rs151015596 | T>C | missense | 4/614 | validated | - |
| *TTF2* | 37232 | chr1:117633170 | rs148036415 | G>A | missense | 5/616 | validated | - |
| *TTLL2* | 17351 | chr6:167753792 | rs146306345 | A>G | missense | 0/614 | validated | validated |
| *TTLL2* | 17122 | chr6:167754551 | rs144554559 | A>G | missense | 0/614 | validated | - |
| *TTLL9* | 17122 | chr20:30497577 | - | G>A | missense | 0/614 | - | validated |
| *TUBAL3* | 17351 | chr10:5437414 | rs141364432 | C>G | missense | 3/614 | validated | - |
| *TULP3* | 7435 | chr12:3047365 | rs34246393 | G>C | missense | 2/614 | validated | - |
| *TXLNB* | 7870 | chr6:139598010 | rs150932533 | G>A | missense | 0/614 | validated | - |
| *UACA* | 37024 | chr15:70959422 | rs140913353 | C>A | missense | 0/614 | validated | - |
| *UBXN2A* | 17342 | chr2:24205852 | rs141503335 | C>T | missense | 0/616 | validated | validated |
| *UIMC1* | 18074 | chr5:176409574 | rs13167812 | G>A | missense | 13/614 | validated | - |
| *UQCC* | 7503 | chr20:33891824 | rs41290916 | C>T | missense | 0/614 | validated | - |
| *URB1* | 18074 | chr21:33711126 | rs141550612 | G>A | missense | 8/608 | validated | - |
| *USP35* | 17342 | chr11:77921348 | rs75370284 | G>A | missense | 11/614 | validated | - |
| *UTRN* | 3836 | chr6:144811319 | rs77066116 | A>G | missense | 5/614 | - | validated |
| *UTRN* | 7936 | chr6:144854342 | rs35676466 | G>A | missense | 8/614 | - | validated |
| *VEGFC* | 7531 | chr4:177650866 | rs41278571 | C>T | missense | 7/614 | validated | - |
| *VIL1* | 7713 | chr2:219299328 | - | A>C | missense | 0/616 | validated | - |
| *VPS13B* | 17678 | chr8:100832259 | rs28940272 | A>G | missense | 2/614 | validated | validated |
| *WDR36* | 37674 | chr5:110436345 | rs142088179 | A>C | missense | 9/614 | validated | - |
| *WDR4* | 17351 | chr21:44270267 | rs146736520 | C>G | missense | 0/612 | - | validated |
| *WDR53* | 37994 | chr3:196287884 | - | A>G | missense | 0/616 | - | validated |
| *WDR60* | 37674 | chr7:158663939 | - | A>G | missense | 1/596 | validated | - |
| *WDR93* | 7728 | chr15:90245033 | rs77441492 | G>A | missense | 17/614 | validated | - |
| *YSK4* | 37037 | chr2:135756467 | rs141899370 | G>A | missense | 0/616 | validated | - |
| *ZAN* | 17122 | chr7:100346094 | rs117406702 | G>A | splicing | 24/608 | validated | - |
| *ZEB2* | 7531 | chr2:145157478 | - | A>T | missense | 0/616 | validated | - |
| *ZMYM4* | 7531 | chr1:35847032 | rs34924462 | G>A | missense | 24/616 | validated | - |
| *ZMYND17* | 37425 | chr10:75184444 | rs11591720 | A>G | missense | 11/614 | validated | validated |
| *ZMYND17* | 17122 | chr10:75187395 | rs150492078 | G>A | missense | 1/614 | validated | validated |
| *ZNF142* | 37150 | chr2:219521110 | rs61733649 | C>T | missense | 3/616 | validated | - |
| *ZNF16* | 37994 | chr8:146157104 | rs145028977 | C>G | missense | 13/614 | validated | validated |
| *ZNF187* | 7745 | chr6:28240525 | - | C>T | missense | 1/588 | validated | - |
| *ZNF197* | 17351 | chr3:44683454 | rs148794995 | G>C | missense | 1/616 | validated | - |
| *ZNF208* | 17351 | chr19:22154331 | - | A>G | missense | 0/612 | - | validated |
| *ZNF208* | 17351 | chr19:22155362 | - | G>C | missense | 2/614 | - | validated |
| *ZNF229* | 7506 | chr19:44932747 | - | C>T | missense | 0/614 | validated | - |
| *ZNF266* | 7590 | chr19:9524715 | rs78504948 | A>G | missense | 7/614 | validated | - |
| *ZNF276* | 17678 | chr16:89793731 | rs17719249 | C>T | missense | 7/614 | validated | - |
| *ZNF280D* | 7658 | chr15:56923895 | rs61741526 | C>A | missense | 16/614 | validated | - |
| *ZNF304* | 37037 | chr19:57863052 | rs117111481 | T>C | missense | 16/604 | validated | - |
| *ZNF323* | 7745 | chr6:28297279 | rs148156232 | C>T | missense | 4/614 | validated | - |
| *ZNF559* | 17351 | chr19:9452698 | rs74575837 | G>A | missense | 18/614 | validated | - |
| *ZNF571* | 17342 | chr19:38055957 | rs61731963 | C>T | missense | 3/614 | validated | - |
| *ZNF646* | 7506 | chr16:31091390 | rs35376811 | C>T | missense | 6/608 | validated | - |
| *ZNF653* | 7531 | chr19:11598224 | rs74552618 | C>T | missense | 12/596 | validated | - |
| *ZNF805* | 37425 | chr19:57765985 | - | G>A | missense | 0/614 | validated | - |
| *ZNF831* | 7728 | chr20:57766673 | - | C>T | missense | 4/608 | validated | - |
| *ZNF836* | 37037 | chr19:52658231 | rs61744685 | C>T | missense | 4/614 | validated | - |

| **Supporting Table 5 - Genes with Damaging, Validated Variants in More Than One Family** | | | | | | |
| --- | --- | --- | --- | --- | --- | --- |
|  |  |  |  |  |  |  |
| **Gene** | **Family** | **Position (Hg19)** | **dbSNP** | **Nucleotide** | **Amino Acid** | **HIHG control chromosomes** |
| *ADRA1A* | 37037 | chr8:26721888 | rs2229125 | A>C | Ile200Ser | 13/610 |
|  | 7531 | chr8:26722027 | rs61757009 | A>C | Ser154Ala | 2/612 |
| *ALS2CL* | 17545 | chr3:46712490 | rs77367607 | G>A | Ser949Phe | 20/614 |
|  | 17122 | chr3:46719860 | rs140347863 | T>C | Asn549Ser | 0/612 |
| *ANKRD30A* | 7435 | chr10:37419292 | rs116939015 | G>T | Glu110Stop | 12/612 |
|  | 37232 | chr10:37508345 | - | G>T | Met1179Ile | 0/612 |
| *C17orf66* | 37150 | chr17:34186056 | - | C>G | Glu259Gln | 0/614 |
|  | 17678 | chr17:34192351 | rs141724302 | G>A | Pro63Leu | 6/614 |
| *C1orf168* | 3836, 7936 | chr1:57254697 | rs41305876 | G>A | Leu290Phe | 5/616 |
| *CCDC74A* | 17351 | chr2:132287879 | - | G>A | Gly110Glu | 0/598 |
|  | 37037 | chr2:132290912 | rs140033985 | G>A | Glu360Lys | 12/614 |
| *CEP290* | 37425 | chr12:88472996 | rs61941020 | C>T | Arg1746Gln | 3/610 |
|  | 37117 | chr12:88508258 | rs79705698 | T>C | Asp664Gly | 15/612 |
| *COL6A3* | 18074 | chr2:238253016 | rs151079701 | G>A | Arg2549Trp | 0/616 |
|  | 17478 | chr2:238271991 | rs146546544 | G>A | Arg1990Trp | 0/616 |
| *CSMD1* | 37117 | chr8:2965294 | - | G>C | Pro2123Ala | 2/614 |
|  | 17122 | chr8:3253832 | - | C>T | Gly689Asp | 0/604 |
| *DMXL1* | 17678 | chr5:118505962 | rs139856633 | C>T | Arg1826Cys | 1/614 |
|  | 37674 | chr5:118506652 | - | C>T | Arg2056Cys | 0/614 |
| *DNAJC30* | 7506, 17351 | chr7:73097713 | rs61751896 | G>A | Pro14Leu | 25/612 |
| *FAT1* | 37037 | chr4:187518041 | rs72716244 | T>C | Asp4218Gly | 11/610 |
|  | 17545 | chr4:187549364 | rs111886222 | G>A | Thr1585Met | 5/614 |
|  | 17545 | chr4:187557908 | rs113970444 | C>T | Arg1268Gln | 5/608 |
| *FBN3* | 7590 | chr19:8175770 | rs17160194 | T>A | Asn1431Ile | 0/614 |
|  | 37674 | chr19:8183871 | rs35579498 | G>A | Arg1083Trp | 25/614 |
| *FBXO30* | 7658 | chr6:146121343 | - | C>G | Glu713Gln | 0/614 |
|  | 37994 | chr6:146126163 | rs150645956 | G>A | Thr460Ile | 1/614 |
| *GJA8* | 7663, 37232 | chr1:147380740 | rs138140155 | A>G | Asn220Asp | 6/616 |
| *KCNB2* | 3836 | chr8:73849256 | rs144823279 | C>T | Pro556Ser | 0/614 |
|  | 17678 | chr8:73850175 | - | A>G | Asn862Ser | 0/614 |
| *KIAA1614* | 7713, 37232 | chr1:180905263 | rs17302207 | C>T | Arg740Trp | 23/614 |
| *MDP1* | 7531, 37674 | chr14:24683304 | rs145254894 | C>A | Gly153Trp | 9/614 |
| *MYO9A* | 17122, 17302 | chr15:72189952 | rs80283650 | T>A | Asn1631Ile | 13/614 |
| *MYOM1* | 37232 | chr18:3135575 | rs115382168 | T>C | Thr727Ala | 0/598 |
|  | 17351 | chr18:3135644 | rs149528866 | C>T | Glu704LySer | 3/604 |
|  | 17351 | chr18:3215083 | - | T>C | Ser47Gly | 1/588 |
| *NINL* | 17122, 37425 | chr20:25439036 | rs41310175 | G>A | Arg1276Cys | 21/614 |
| *OAS3* | 7936, 17545 | chr12:113403675 | rs61942233 | C>T | Arg844Stop | 11/602 |
| *OR51L1* | 7435, 37674 | chr11:5020933 | rs61729748 | A>T | Asn241Tyr | 15/614 |
| *OTOF* | 17342 | chr2:26703734 | rs55676840 | C>T | Val575Met | 4/610 |
|  | 17545 | chr2:26750775 | rs150132765 | G>A | Pro51Leu | 1/616 |
| *POLE* | 7637 | chr12:133240613 | - | C>T | Ala895Thr | 0/614 |
|  | 17678 | chr12:133252760 | - | A>C | Ser314Ala | 0/614 |
| *POLM* | 7506, 17351 | chr7:44118394 | rs28382644 | C>G | Gly220Ala | 6/614 |
| *RDH11* | 17122, 37037 | chr14:68159269 | rs80140987 | C>T | Glu79Lys | 11/614 |
| *SLC26A3* | 37037 | chr7:107427322 | rs34407351 | A>C | Cys307Trp | 26/614 |
|  | 17122 | chr7:107434192 | - | A>T | Leu89Gln | 0/614 |
| *SLIT3* | 17342, 18074 | chr5:168180047 | rs34260167 | C>T | Ser629Asn | 7/614 |
| *SOS2* | 37024 | chr14:50655307 | rs61755579 | C>T | Ala208Thr | 13/614 |
|  | 37024 | chr14:50655357 | rs72681869 | G>C | Pro191Arg | 6/614 |
|  | 37425 | chr14:50655380 | rs137961578 | C>G | Leu183Phe | 0/614 |
| *SPATA12* | 17351, 17545 | chr3:57108101 | rs76587478 | C>T | Pro127Ser | 9/616 |
| *SPNS3* | 37150 | chr17:4349389 | rs147085241 | C>T | Ser150Leu | 0/614 |
|  | 7658 | chr17:4391185 | rs149163440 | C>G | Pro512Arg | 3/614 |
| *STXBP5* | 37994 | chr6:147635108 | rs144099092 | C>G | Leu412Val | 2/614 |
|  | 7623 | chr6:147636753 | rs148830578 | A>G | Tyr502Cys | 2/614 |
| *TBC1D2B* | 17122, 17302 | chr15:78337303 | rs61732053 | C>T | Ala208Thr | 25/608 |
| *TET2* | 7531, 7713 | chr4:106156163 | rs61744960 | G>A | Gly355Asp | 29/614 |
| *TMC7* | 37534 | chr16:19020725 | rs118019760 | A>C | Lys100Thr | 4/614 |
|  | 37232 | chr16:19033065 | - | C>T | Thr192Met | 0/614 |
| *TPMT* | 7713, 39312 | chr6:18139228 | rs1800460 | C>T | Ala154Thr | 23/614 |
| *TRIM55* | 17678 | chr8:67047224 | rs141724668 | G>A | splice site | 0/614 |
|  | 37425 | chr8:67067937 | - | C>T | Thr535Ile | 1/614 |
| *TRPM6* | 7590 | chr9:77367203 | rs55679040 | T>C | Gln1663Arg | 10/614 |
|  | 7506 | chr9:77454973 | rs150874152 | C>T | Gly171Arg | 1/614 |
| *TTLL2* | 17351 | chr6:167753792 | rs146306345 | A>G | His135Arg | 0/614 |
|  | 17122 | chr6:167754551 | rs144554559 | A>G | Asn388Ser | 0/614 |
| *UTRN* | 3836 | chr6:144811319 | rs77066116 | A>G | Gln1416Arg | 5/614 |
|  | 7936 | chr6:144854342 | rs35676466 | G>A | Gly2060Asp | 8/614 |
| *ZMYND17* | 37425 | chr10:75184444 | rs11591720 | A>G | Leu417Pro | 11/614 |
|  | 17122 | chr10:75187395 | rs150492078 | G>A | Ser118Phe | 1/614 |

| **Supporting Table 6 - Families with Multiple Damaging, Validated Variants in the Same Gene** | | | | | | |
| --- | --- | --- | --- | --- | --- | --- |
|  |  |  |  |  |  |  |
| **Gene** | **Family** | **Position (Hg19)** | **dbSNP** | **Nucleotide** | **Amino Acid** | **HIHG control chromosomes** |
| *ABHD14A* | 17351 | chr3:52011912 | rs17849626 | G>A | Arg32Gln | 25/614 |
|  |  | chr3:52014897 | rs61729088 | C>G | Arg227Gly | 2/616 |
| *BPHL* | 7531 | chr6:3140676 | - | G>A | Gly224Ser | 0/614 |
|  |  | chr6:3152781 | rs140507260 | A>G | Asn266Thr | 0/614 |
| *BTN2A2* | 37425 | chr6:26384060 | rs73736234 | C>T | Ala4Val | 3/614 |
|  |  | chr6:26385263 | rs57038103 | C>G | Pro39Ala | 2/614 |
| *C2orf85* | 18074 | chr2:242814639 | rs75447317 | T>C | Leu311Pro | 5/610 |
|  |  | chr2:242815059 | rs28368764 | C>T | Ala451Val | 6/612 |
| *DNAH10* | 7936 | chr12:124356105 | - | G>T | Ala2463Ser | 0/610 |
|  |  | chr12:124401027 | rs61745785 | C>A | Asp3464Glu | 5/608 |
| *FAT1* | 17545 | chr4:187549364 | rs111886222 | G>A | Thr1584Met | 5/614 |
|  |  | chr4:187557908 | rs113970444 | C>T | Arg1268Gln | 5/608 |
| *FYCO1* | 17351 | chr3:46003735 | rs41289620 | C>T | Arg1140Gln | 5/616 |
|  |  | chr3:46008983 | rs149507450 | G>A | Arg615Trp | 5/616 |
| *MDC1* | 37425 | chr6:30679963 | rs2844707 | A>C | Ser586Ala | 3/612 |
|  |  | chr6:30680968 | rs2517560 | C>T | Glu251Lys | 2/612 |
| *MYH8* | 7590 | chr17:10298599 | rs139344968 | C>T | Asp1605Asn | 1/614 |
|  |  | chr17:10298628 | rs145711576 | C>G | Arg1595Thr | 1/614 |
| *MYOM1* | 17351 | chr18:3135644 | rs149528866 | C>T | Glu704LySer | 3/604 |
|  |  | chr18:3215083 | - | T>C | Ser47Gly | 1/588 |
| *OFCC1* | 7606 | chr6:9809860 | - | C>T | Arg538Gln | 0/602 |
|  |  | chr6:9900660 | rs148761621 | C>A | Glu204Stop | 0/614 |
| *PRICKLE1* | 37232 | chr12:42862463 | rs61924369 | C>T | Glu185Lys | 0/614 |
|  |  | chr12:42864125 | - | C>G | Val57Leu | 0/614 |
| *SOS2* | 37024 | chr14:50655307 | rs61755579 | C>T | Ala208Thr | 13/614 |
|  |  | chr14:50655357 | rs72681869 | G>C | Pro191Arg | 6/614 |
| *ZNF208* | 17351 | chr19:22154331 | - | A>G | Tyr1169H | 0/612 |
|  |  | chr19:22155362 | - | G>C | Ala825Gly | 2/614 |

| **Supporting Table 7 - Damaging, Validated Variants in Genes Previously Implicated in ASD or Other Disorders** | | | | | | | | |
| --- | --- | --- | --- | --- | --- | --- | --- | --- |
|  |  |  |  |  |  |  |  |  |
| **Gene** | **Family** | **Position (Hg19)** | **dbSNP** | **Nucleotide** | **Amino Acid** | **HIHG control chromosomes** | **Disorder*** | **References** |
| *ABCA13* | 7936 | chr7:48273776 | - | C>T | - | 0/610 | ASD, BD, MD, SZ | Knight, et al, 2009, Neale, et al, 2012 |
| *ABHD14A* | 17351 | chr3:52011912 | rs17849626 | G>A | Arg32Gln | 25/614 | ASD | Casey, et al, 2012 |
|  | 17351 | chr3:52014897 | rs61729088 | C>G | Arg227Gly | 2/616 | ASD | Casey, et al, 2012 |
| *ABI3BP* | 18074 | chr3:100585792 | rs113364496 | C>T | Glu314Lys | 9/586 | ASD | Sanders, et al, 2012 |
| *AGAP1* | 17478 | chr2:236659033 | rs143378661 | G>A | Asp192Asn | 0/616 | ASD | Wassink, et al, 2005 |
| *AP4M1* | 17122 | chr7:99702946 | - | C>T | Arg271Cys | 0/614 | ID | Abou Jamra, et al, 2011 |
| *CDH9* | 17351 | chr5:26885797 | rs34490509 | T>C | Glu603Gly | 0/614 | ASD | Ma, et al, 2009, Wang, et al, 2009 |
| *CEP290* | 37425 | chr12:88472996 | rs61941020 | C>T | Arg1746Gln | 3/610 | ASD, ID | Coppieters, et al, 2010 |
|  | 37117 | chr12:88508258 | rs79705698 | T>C | Asp664Gly | 15/612 | ASD, ID | Coppieters, et al, 2010 |
| *CLCN2* | 37994 | chr3:184076909 | rs151257924 | C>T | Arg25Gln | 0/616 | E | Everett, et al, 2007 |
| *CNTN5* | 17342 | chr11:100168410 | rs141228828 | T>A | Leu790Ile | 3/612 | ADHD, ASD, SZ | Glessner, et al, 2010, Lionel, et al, 2011, van Daalen, et al, 2011 |
| *CPZ* | 37232 | chr4:8613762 | rs147588134 | G>T | Lyn412Asn | 2/616 | ASD | Sanders, et al, 2012 |
| *CSMD1* | 37117 | chr8:2965294 | - | G>C | Pro2262Ala | 2/614 | ASD, SZ | Havik, et al, 2011 |
|  | 17122 | chr8:3253832 | - | C>T | Gly827Asp | 0/604 | ASD, SZ | Havik, et al, 2011 |
| *DNAH9* | 7590 | chr17:11840819 | rs144547132 | G>T | Gly526Cys | 0/614 | ASD | Sanders, et al, 2012 |
| *DLGAP2* | 37117 | chr8:1624708 | - | G>C | Ala737Pro | 0/594 | ASD | Marshall, et al, 2008 |
| *F13A1* | 37994 | chr6:6251120 | rs3024477 | T>A | Try205Phe | 20/614 | ASD, ID | Hu, et al, 2006 |
| *FAT1* | 37037 | chr4:187518041 | rs72716244 | T>C | Asp4218Gly | 11/610 | ASD, BD | Blair, et al, 2006, Neale, et al, 2012 |
|  | 17545 | chr4:187549364 | rs111886222 | G>A | Thr1585Met | 5/614 | ASD, BD | Blair, et al, 2006, Neale, et al, 2012 |
|  | 17545 | chr4:187557908 | rs113970444 | C>T | Arg1268Gln | 5/608 | ASD, BD | Blair, et al, 2006, Neale, et al, 2012 |
| *FBXO40* | 37425 | chr3:121340955 | rs148776238 | G>A | Ala227Thr | 12/616 | ASD | Glessner, et al, 2009 |
| *FRK* | 37674 | chr6:116325108 | rs34064900 | G>A | Ser133Leu | 6/616 | ASD, ID | Wang, et al, 2009 |
| *GRIN3B* | 37024 | chr19:1008645 | rs78914045 | C>A | Ala832Glu | 5/600 | ASD | O'Roak, et al, 2012 |
| *HTR7* | 17302 | chr10:92509055 | rs114969659 | G>A | Pro279Leu | 0/614 | ASD | Lassig, et al, 1999 |
| *IQGAP2* | 7637 | chr5:75960968 | rs34968964 | G>C | Glu883Gln | 4/614 | ASD | O'Roak, et al, 2012 |
| *ITSN2* | 7435 | chr2:24435599 | rs41281481 | G>A | Arg1337Trp | 30/616 | ASD | Sanders, et al, 2012 |
| *JARID2* | 37994 | chr6:15496930 | rs150448457 | C>T | Arg492Cys | 6/602 | ASD, SZ | Liu, et al, 2009, Weiss, et al, 2009 |
| *KIAA1949* | 37425 | chr6:30652729 | rs2213944 | G>A | Pro356Leu | 3/612 | ASD | Neale, et al, 2012 |
| *LLGL1* | 17678 | chr17:18145552 | rs149387516 | C>A | Ser985Arg | 0/614 | ASD | Sanders, et al, 2012 |
| *MYO1A* | 17678 | chr12:57431366 | rs148808080 | C>T | Gly674Asp | 0/614 | ASD | O'Roak, et al, 2011 |
| *MYOM2* | 7663 | chr8:2050520 | - | G>A | Val895Met | 1/614 | ASD | Neale, et al, 2012 |
| *NRCAM* | 17342 | chr7:107822355 | rs150373689 | G>A | Arg853Cys | 1/614 | ASD | Marui, et al, 2009 |
| *NRXN2* | 17478 | chr11:64453180 | rs146308270 | C>T | Val364Ile | 1/614 | ASD | Gauthier, et al, 2011 |
| *OFCC1* | 7606 | chr6:9809860 | - | C>T | Arg538Gln | 0/602 | TS | Sundaram, et al, 2011 |
|  | 7606 | chr6:9900660 | rs148761621 | C>A | Glu204Stop | 0/614 | TS | Sundaram, et al, 2011 |
| *PLCD1* | 37994 | chr3:38051211 | rs75495843 | G>A | Ser481Leu | 14/614 | ASD | O'Roak, et al, 2011 |
| *PRICKLE1* | 37232 | chr12:42862463 | rs61924369 | C>T | Glu185Lys | 0/614 | E | Bassuk, et al, 2008, Tao, et al, 2011 |
|  | 37232 | chr12:42864125 | - | C>G | Val57Leu | 0/614 | E | Bassuk, et al, 2008, Tao, et al, 2011 |
| *PRSS38* | 37534 | chr1:228004966 | rs61826983 | C>A | Ala123Asp | 16/616 | ASD | Connolly, et al, 2013 |
| *PRUNE2* | 7590 | chr9:79465455 | rs41304230 | C>T | Asp90Asn | 6/614 | ASD | Vaags, et al, 2012 |
| *PTPRC* | 7713 | chr1:198668761 | rs41269905 | G>C | Asp123His | 9/616 | ASD | Pinto, et al, 2010 |
| *RELN* | 17342 | chr7:103234202 | rs55689103 | C>T | Gly1280Glu | 20/614 | ASD, ID | Neale, et al, 2012 |
| *SLC8A3* | 37425 | chr14:70633411 | rs41286548 | C>T | Gly577Ser | 13/614 | ASD | Sanders, et al, 2012 |
| *SLIT3* | 17342, 18074 | chr5:168180047 | rs34260167 | C>T | Ser629Asn | 7/616 | MD | Glessner, et al, 2010 |
| *SMARCC1* | 17545 | chr3:47777540 | - | A>G | Asp187Gly | 0/616 | ASD | Neale, et al, 2012 |
| *STXBP5* | 37994 | chr6:147635108 | rs144099092 | A>G | Leu412Val | 2/614 | ASD | Davis, et al, 2009 |
|  | 7623 | chr6:147636753 | rs148830578 | T>A | Try502Cys | 2/614 | ASD | Davis, et al, 2009 |
| *SYNE1* | 17122 | chr6:152779933 | rs34610829 | G>A | Arg850Cys | 14/614 | ASD, BD, MD | Green, et al, 2012, Yu, et al, 2013 |
| *SYNE2* | 37994 | chr14:64676751 | rs36215895 | C>T | Thr6211Met | 1/612 | ASD | Neale, et al, 2012 |
| *SYN1* | 37674 | chr23:47466545 | - | C>T | Glu144Lys | 0/614 | ASD, E | Garcia, et al, 2004, Fassio, et al, 2011 |
| *TOPORS* | 37232 | chr9:32542278 | rs17857515 | T>C | Asn749Asp | 5/614 | ASD | Neale, et al, 2012 |
| *TSPYL5* | 37425 | chr8:98289238 | rs151015596 | T>C | Ser279Gly | 4/614 | ASD | Sanders, et al, 2012 |
| *TTF2* | 37232 | chr1:117633170 | rs148036415 | G>A | Arg838His | 5/616 | ASD | Neale, et al, 2012 |
| *UIMC1* | 18074 | chr5:176409574 | rs13167812 | G>A | Arg15Trp | 13/614 | ASD | O'Roak, et al, 2012 |
| *VPS13B* | 17678 | chr8:100832259 | rs28940272 | A>G | Asn2968Ser | 2/612 | ASD | Kolehmainen, et al, 2003, Howlin, et al, 2005 |
| *WDR4* | 17351 | chr21:44270267 | rs146736520 | C>G | Glu377Asp | 0/612 | ASD | Pereira, et al, 2009 |
| *WDR60* | 37674 | chr7:158663939 | - | A>G | Asp59Gly | 1/596 | ID, SZ | Tyson, et al, 2005, Kirov, et al, 2009 |
| *ADHD - attention deficit hyperactivity disorder, ASD - autism spectrum disorder, BD - bipolar disorder, E - epilepsy, ID -intellectual disability, MD - major depression, SZ - schizophrenia, TS - Tourette syndrome | | | | | | | | |
